# Addressing the affordability of cancer drugs: using deliberative public engagement to inform health policy

**DOI:** 10.1186/s12961-019-0411-8

**Published:** 2019-02-07

**Authors:** Colene Bentley, Stuart Peacock, Julia Abelson, Michael M. Burgess, Olivier Demers-Payette, Holly Longstaff, Laura Tripp, John N. Lavis, Michael G. Wilson

**Affiliations:** 1 Canadian Centre for Applied Research in Cancer Control (ARCC), 675 West 10th Avenue, Vancouver, British Columbia V5Z 1L3 Canada; 2 Department of Health Research Methods, Evidence and Impact, McMaster University, 1280 Main Street West (CRL 203), Hamilton, Ontario L8S 4K1 Canada; 3 School of Population and Public Health, University of British Columbia, 239 RHS, 1088 Discovery Avenue, Kelowna, British Columbia V1V 1V7 Canada; 4 Institute National d’Excellence en Santé et en Services Sociaux (INESSS), 2021 avenue Union, 12th Floor, bureau 1200, Montreal, Quebec H3A 2S9 Canada; 5 Engage Associates Consulting Firm, Vancouver, British Columbia Canada; 6 McMaster Health Forum, 1280 Main Street West, MML-417, Ontario, Hamilton L8S 4L6 Canada

**Keywords:** Public engagement, priority-setting, cancer, Canada

## Abstract

**Background:**

Health system expenditure on cancer drugs is rising rapidly in many OECD countries given the costly new treatments and increased rates of use due to a growing and ageing population. These factors put considerable strain on the sustainability of health systems worldwide, sparking public debate among clinicians, pharmaceutical companies, policy-makers and citizens on issues of affordability and equity. We engaged Canadians through a series of deliberative public engagement events to determine their priorities for making cancer drug funding decisions fair and sustainable in Canada’s publicly financed health system.

**Methods:**

An approach to deliberation was developed based on the McMaster Health Forum’s citizen panels and the established Burgess and O'Doherty model of deliberative public engagement. Six deliberations were held across Canada in 2016. Transcripts were coded in NVivo and analysed to determine where participants’ views converged and diverged. Recommendations were grouped thematically.

**Results:**

A total of 115 Canadians participated in the deliberative events and developed 86 recommendations.

Recommendations included the review and regular re-review of approved drugs using ‘real-world’ evidence on effectiveness and cost-effectiveness; prioritisation of treatments that restore patients’ independence, mental health and general well-being; ensuring that decision processes, results and their rationales are transparent; and commitment to people with similar needs receiving the same care regardless of where in Canada they live.

**Conclusions:**

The next steps for policy-makers should be to develop mechanisms for (1) re-reviewing effectiveness and cost-effectiveness data for all cancer drugs; (2) making disinvestments in cancer drugs that satisfy requirements relating to grandfathering and compassionate access; (3) ensuring fair and equitable access to cancer drugs for all Canadians; and (4) fostering a pan-Canadian approach to cancer drug funding decisions.

**Electronic supplementary material:**

The online version of this article (10.1186/s12961-019-0411-8) contains supplementary material, which is available to authorized users.

## Background

Health system expenditure on cancer drugs is rising rapidly in many OECD countries [1], sparking widespread debate in the public arena, as well as among clinicians, pharmaceutical companies and policy-makers worldwide. In Canada, the situation is no different. The affordability of cancer drugs is particularly concerning for Canada, which is third only to the United States and Switzerland in terms of dollars spent on pharmaceuticals per capita [2].

Several factors are driving up expenditures on cancer drugs, including prices for new cancer drugs increasing dramatically [3, 4], the use of systemic therapies in more patients [5, 6], the sharp rise in incident cases (due to, among other reasons, patients living longer) [7, 8] and the acceleration in the number of new cancer drugs (often with higher daily drug costs and longer duration of treatment) [9]. Together, these factors put considerable strain on the affordability and sustainability of Canada’s publicly financed provincial and territorial health systems.

Canada’s pan-Canadian Oncology Drug Review, which is part of the Canadian Agency for Drugs and Technologies in Health (CADTH), utilises a national review process for new cancer drugs. It reviews the clinical and economic evidence (among other inputs) about each new cancer drug and makes funding recommendations to Canada’s provincial and territorial governments, which are responsible for healthcare funding decisions [10]. However, its recommendations do not address the economic and ethical challenges that provincial and territorial policy-makers face about how to cover increasingly expensive cancer drugs given limited budgets and competing health priorities.

There is relatively little guidance on if, when and how to consult the public on priority-setting and resource allocation decisions [11, 12]. In recent years, deliberative forms of public engagement have emerged as robust approaches to public consultation on many health policy initiatives [13, 14]. Deliberative public engagement brings members of the public together in a process of learning and dialogic exchange focused on collective problem-solving that is not consensus driven [13, 15]. In Canada, deliberative engagements have been used to advise provincial Ministries of Health on technology assessment [16], health services [17] and biobanks [18], among many other topics.

While relatively little is known about what Canadian clinicians and policy-makers value concerning different cancer programmes and interventions and their outcomes, even less is known about Canadians’ values on these matters [19]. To address this knowledge gap, we explored the concerns, perspectives and values of Canadians on the topic of cancer drug funding, and brought their recommendations to the attention of policy-makers who must confront issues of affordability, sustainability and fairness in their healthcare jurisdictions.

## Methods

Five 2-day citizen panels – or deliberative public engagement events – were convened in four Canadian provinces (Saskatchewan, Ontario, Quebec and Nova Scotia) between April and June 2016. Provinces were selected to reflect different geographic regions, cancer delivery programmes and drug budgets across Canada. Two of the five panels were held in Quebec (one in English and one in French). A sixth ‘pan-Canadian’ panel was convened in October 2016 (and, as described below, drew on participants from the five panels as well as from an earlier, similar provincial event in British Columbia in 2014).

The panel design combined the strengths of two well-established deliberative public engagement approaches, namely that of the McMaster Health Forum (www.mcmasterhealthforum.org/citizens/citizen-briefs-and-panels) and the ‘mini-public’ approach of Burgess, Longstaff and O’Doherty [20]. These approaches support citizens from a variety of backgrounds and perspectives to participate in meaningful dialogic exchange, where their deliberations are directed towards collective problem-solving while highlighting the acceptable trade-offs of a given policy initiative. Deliberative approaches draw on citizens’ perspectives to help define policy issues and advise on the social and normative aspects of decision-making [17, 21]. Empirical evidence has shown that the public can make coherent and sophisticated recommendations concerning values and health policy [22] and provide substantive knowledge to policy-makers. Deliberative approaches can enhance accountability in government decision-making [23], improve the legitimacy of decisions taken [24] and promote public understanding of complex healthcare issues [17].

### Participant recruitment

The goal of recruitment was to identify a group of 20–25 citizens to participate in each panel who reflected a diversity of life experiences and social perspectives based on the demographics of the province in which each panel was held. An online market research company, AskingCanadians™, was engaged to conduct recruitment. A letter of invitation to participate in the deliberation was e-mailed to a randomly selected group of AskingCanadians™ online panel members. Interested individuals completed an eligibility survey on the following criteria: age, income, education, ethno-cultural background, lived experience with chronic disease (as patient or caregiver; type of chronic disease was unspecified) and geographic location within their home province. Individuals were not eligible to participate if they were employees of healthcare organisations or health professionals, employees or those with a direct financial relationship with a tobacco or pharmaceutical company, individuals who had lobbied for health advocacy groups, health policy-makers, including both elected officials and public servants, people who had worked for market research, advertising, public media or public relations firms, or individuals who had previously participated in a citizen panel hosted by one of the research collaborators.

Recruitment for the pan-Canadian event involved randomly selecting 3–5 participants from each of the provincial panels who had expressed their interest in being invited to the pan-Canadian event. AskingCanadians™ oversaw the initial contact with prospective participants from each provincial panel and used the same stratified approach to recruitment.

Participants received a $125 honorarium per 8-hour day for the deliberation and their expenses were covered.

### Deliberation topics, questions and information supports

Deliberation topics and questions were developed in consultation with provincial cancer policy-makers in each of the provinces where the panels were held, and with members of the project’s steering and advisory committees. In keeping with an ‘integrated knowledge translation’ or ‘co-production’ model, the steering committee provided guidance and oversight on the project’s direction. It was comprised of three senior decision-makers in cancer control, two of whom were from pan-Canadian organisations and one was in charge of a provincial drugs budget. The advisory committee included members of the research team with expertise in deliberative public engagement and recruitment methods, research ethics, and institutional oversight. They provided advice to ensure that the project was in compliance with relevant regulations (e.g. informed consent processes, financial reporting, etc.).

The overarching deliberation topics and questions were:

- Provincial panels:
  - What should guide policy decisions about whether to fund new cancer drugs or change the funding provided for existing cancer drugs?
  - What would make cancer drug funding decisions trustworthy?
  - How can we improve existing approaches to decision-making about cancer drug funding?
- Pan-Canadian panel:
  - What are important features of a pan-Canadian approach to making funding decisions about cancer drugs?
  - What are the trade-offs associated with a pan-Canadian approach to making funding decisions about cancer drugs?
  - How might these trade-offs be addressed to produce trustworthy decisions?

Several information sources were developed to support participants’ deliberations. Prior to each event, participants received a link to the video ‘Cancer Dialogues’, which featured two oncologists, a health economist and a patient advocate, each speaking about cancer drug funding from their own perspective (see https://cc-arcc.ca/societal-values-public-engagement-2/). Participants also received a plain-language citizen brief containing relevant research evidence on the topic of cancer drug funding decisions in Canada, how these decisions are made, the drug coverage plans for the province in which the panel was held, and several additional questions to encourage participants to reflect upon the information provided (see Additional file 1 for the citizen brief for the pan-Canadian panel). At each event, a cancer patient representative and an oncologist from the area gave brief remarks and answered questions. The oncologists also had expert knowledge of cancer drug funding processes and budgets locally and/or at the pan-Canadian level. This provided experiential perspectives and local decision-making context for panel participants.

### Structure of deliberations

All panels followed the same format. On Day 1, participants heard from expert speakers and asked them questions, viewed the video, and deliberated and made recommendations on Topic 1. On Day 2, participants deliberated on Topics 2 and 3 and made recommendations on them.

Panel discussions were led by trained facilitators in large and small group sessions. Each deliberation topic was discussed first in small group sessions, followed by collective deliberation and making recommendations in the large group.

Participants negotiated all recommendation statements. Once a recommendation was formulated, voting was used to gauge the degree of collective support for it, and as a technique to identify points of disagreement or tension that could be recorded and explored more fully. Participants who abstained or voted against a recommendation were prompted to explain their views.

At least one, and often three, principal investigators attended each panel to answer questions of clarification from participants, observe the deliberations first hand and support the consistency of methods across the events.

### Data collection and analysis

All events were audio recorded and transcribed. The integration of recommendations across panels was approached through constant comparison and consultation with transcripts to ensure appropriate interpretation. Differences across regional events and from the broader focus of the pan-Canadian event were considered and reflected in the description of the themes.

Transcript analysis began with a detailed review of the recommendations within their provincial or pan-Canadian context and through a comparative lens. This initial review by the study team led to the identification of a more thematically organised set of categories that aligned with the deliberative topics and questions, and which would provide helpful guidance to policy-makers. Within each thematic category, high-level agreement and divergent perspectives within and across the panel recommendations were identified. A more thorough analysis of the event transcripts was then undertaken to assess how concepts and terms were understood across events and to further characterise the themes. Two qualitative researchers (CB, ODP) who also attended multiple panels analysed the transcripts, which were entered into NVivo 11 software. Weekly sessions were held to review the concepts and terms within each deliberative context. The study team also met weekly to provide feedback on the interpretation of the findings across panels.

### Ethics approval

The project was approved by the Hamilton Integrated Research Ethics Board (#13-369) and the University of British Columbia-British Columbia Cancer Research Ethics Board (#H16-00623). All participants signed a written informed consent form prior to the event.

## Results

A total of 115 citizens participated in the deliberative events, with 20–25 citizens per panel (Table 1).

**Table 1:** Participant demographics

	Provincial panels							Pan-Canadian
	Provincial total	Percentage of total	Hamilton	Halifax	Montreal (English)	Saskatoon	Montreal (French)	
Participants	115		25	24	20	21	25	24
Sex								
Male	53	46%	12	13	8	9	11	13
Female	62	54%	13	11	12	12	14	11
Age, years								
18–24	4	3%	2	0	0	1	1	0
25–34	21	18%	4	6	3	5	3	5
35–49	29	25%	10	6	2	5	6	3
50–64	34	30%	6	5	11	5	7	11
65+	27	23%	3	7	4	5	8	5
Geography								
Urban area	56	49%	13	11	9	10	13	14
Suburban area	44	38%	10	10	8	6	10	7
Rural area	15	13%	2	3	3	5	2	3
Income								
Less than $20,000	11	10%	2	1	3	4	1	3
Between $20,000 and $34,999	25	22%	4	9	1	4	7	5
Between $35,000 and $49,999	15	13%	3	3	4	1	4	4
Between $50,000 and $79,999	28	24%	4	6	6	5	7	4
More than $80,000	14	12%	5	1	1	4	3	4
Prefer not to answer	22	19%	7	4	5	3	3	4
Education								
No certificate	0	0%	0	0	0	0	0	0
High school	14	12%	1	3	2	3	5	0
College or apprenticeship, non-university	23	20%	6	4	5	3	5	9
Some university	23	20%	4	4	4	2	9	3
University or above	55	48%	14	13	9	13	6	12
Children								
With children	62	54%	14	13	11	11	13	17
Without children	53	46%	11	11	9	10	12	7
Ethnicity								
Aboriginal	6	5%	2	1	0	3	0	2
Arab/Middle Eastern	8	7%	2	0	3	2	1	2
Black	10	9%	3	3	0	1	3	3
Chinese	4	3%	1	3	0	0	0	2
Filipino	5	4%	2	1	0	2	0	2
Japanese	3	3%	3	0	0	0	0	2
Korean	0	0%	0	0	0	0	0	0
Latin American	7	6%	3	1	2	0	1	0
South Asian	7	6%	3	2	2	0	0	5
White	58	50%	5	12	12	13	16	5
Other	4	3%	0	1	1	0	2	0
Prefer not to answer	3	3%	1	0	0	0	2	1
Are you currently living with a chronic disease? Do you now or have you in the past cared for someone with one or more chronic diseases?								
Yes/Yes (sufferer/caregiver)	16	14%	3	4	3	3	3	4
No/No (sufferer/caregiver)	48	42%	12	11	6	9	10	5
Yes/No (sufferer/caregiver)	27	23%	6	4	6	5	6	5
No/Yes (sufferer/caregiver)	24	21%	4	5	5	4	6	5
No/no answer (sufferer/caregiver)								2
Yes/no answer (sufferer/caregiver)								3

Across all panels, participants deliberated thoughtfully and respectfully on a range of complex issues related to the affordability, sustainability and fairness of cancer drug funding in Canada. They grasped the core issues under consideration, were able to identify acceptable cost-benefit and equity trade-offs, and provided relevant guidance on making cancer drug funding decisions in Canada.

Participants developed 86 recommendations over the six panels on a range of themes. See Additional file 2 for recommendations from the provincial panels and Additional file 3 for recommendations from the pan-Canadian panel. The themes, described below, draw from the recommendations and transcripts to qualitatively illustrate the areas of strength, convergence and disagreement within and across panels, and to capture important nuances from participants’ discussions. While the themes were supported by all panels, quotations or specific details are drawn from the recommendations of the panel, identified in brackets.

### Theme 1. Evidence and other inputs to support decision-making

There was strong support for clear criteria in drug funding decisions. Participants recommended having “*baseline criteria*” (Nova Scotia) or a “*decision-making tool*” (Saskatchewan) containing specific data elements to guide decision processes so that decisions are based on adequate, identifiable and unbiased information.

The Saskatchewan panel specified the components of a “*decision-making tool*”:

“*The criteria used in the decision-making tool should include but are not limited to cost, quality and quantity of life, side effects, effectiveness, accessibility, incidence and type of cancer, mortality, age, duration of treatment, sustainability of the drug (uninterrupted supply), and ability to compare to other provinces’ decisions.*”

This recommendation reflects the types of information participants across provincial panels felt should guide drug funding decisions. However, the criterion of ‘age’ was explicitly rejected or considered discriminatory at all other provincial panels.

Participants in Ontario supported considering drug costs in light of “*other parts of the health system*” and “*opportunity costs*”. They also considered “*what the public is willing to pay for minimal survival improvements*”, which indicates there may be a threshold beyond which some drugs are not funded.

### Theme 2. Principles to guide funding decisions

**Fig. 1:**
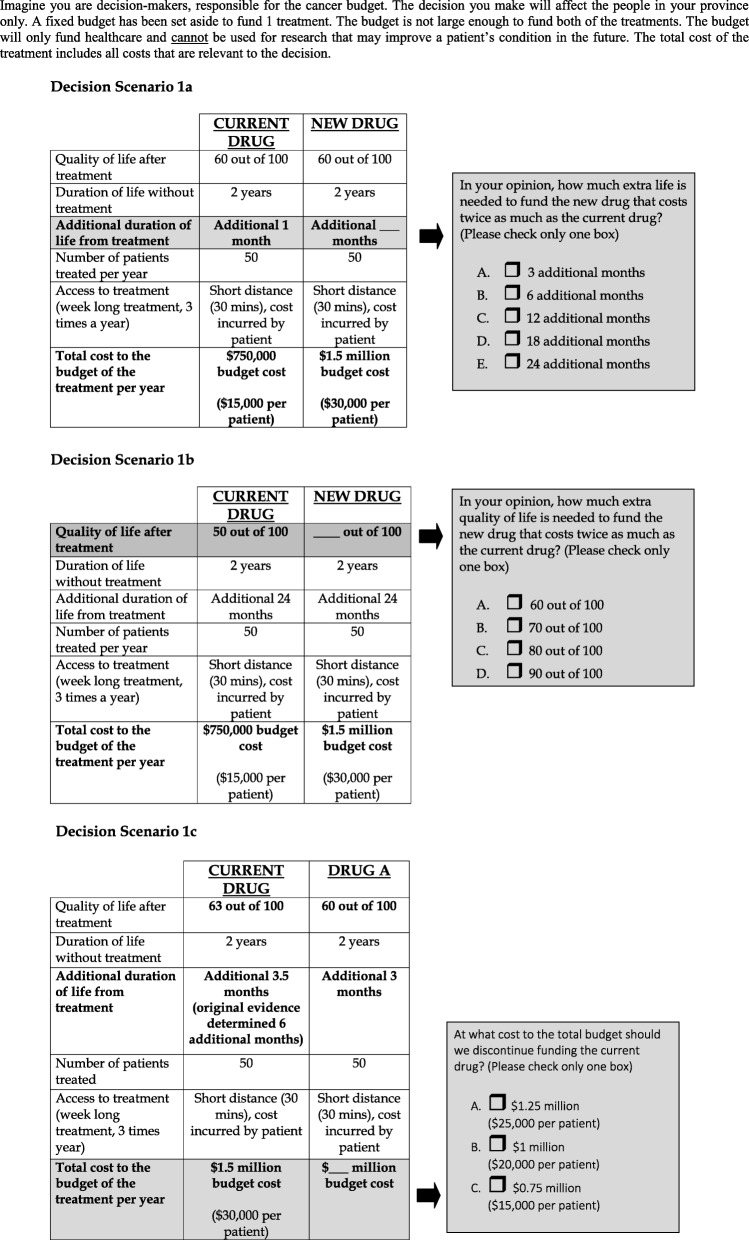
Decision scenarios

Participants articulated the principles they felt should guide funding decisions. At the provincial panels, participants were presented with decision scenarios in which they played the role of policy-makers. The scenarios were intentionally limited to force specific trade-offs between cost and quality or quantity of life, and disinvestment (Fig. 1). Participants’ recommendations reflected what specific health benefit (i.e. improved duration or quality of life) they felt justified the significant additional cost (i.e. a doubling of the cost).

Participants generally thought that modest life extension alone, unless it was sustaining a pre-existing good quality of life, was insufficient justification for approving new or more expensive drugs over alternatives. Most considered quality and length of life interdependently. With respect to funding drugs that extend life, the majority of participants considered doubling the cost, i.e. going from $15,000 to $30,000 per patient, to be a significant cost increase and a worthwhile expenditure only if life was extended by at least 12 months. There was a small degree of support for significant cost being worthwhile for an extension of life of less than 12 months.

Participants in Saskatchewan and at both Quebec events (English and French) specified that, if a cancer drug restored independence for patients and/or improved their mental health, then a higher cost for that drug would be justified.

### Theme 3. Disinvestment and re-review of data and past decisions

There was strong support across all panels for reviewing and reassessing currently approved and used cancer drugs. Participants recommended that reviews be based on clear and consistent principles and on real-world data on the drug’s effectiveness and cost-effectiveness, and that these data be compared with data about other cancer drugs. Participants strongly supported the principle that the health system should fund drugs that are more cost-effective and more clinically effective relative to other cancer drugs. They accepted the need to make tough funding decisions, including stopping or scaling back funding for some currently funded drugs.

Participants supported weighing the costs and benefits of comparable drugs to identify drugs for delisting. They supported replacing cancer drugs based on cost savings for drugs of similar safety and effectiveness. At both Quebec panels (English and French), some participants stipulated that any cost savings from disinvesting in cancer drugs be kept in the cancer drug funding budget envelope and either be re-invested in cancer drugs or in cancer research.

### Theme 4. Ensuring fairness and access

Fairness and equity of access to cancer treatment were important principles across all panels. Participants’ concerns relating to fairness and equity included ensuring access for patients (1) undergoing treatment, (2) living in remote locations and (3) who are members of vulnerable populations.

Recommendations that focused on principles such as ‘compassionate access’ and ‘grandfathering’ reflected the notion that patients receiving treatment should not be disadvantaged by funding decisions and should have the opportunity to continue their treatment. Participants in Saskatchewan, Ontario and Nova Scotia stipulated that, if a drug is delisted, patients should be given the option to stay on the drug, i.e. grandfathering, while alternatives drugs are considered.

Several recommendations focused on improving access in remote regions and on funding for oral drugs because they can be more widely distributed. Personal responsibility for travel-related costs and for oral medications taken in ambulatory care was considered an inequity. Participants also recommended that marginalised populations not be disadvantaged by funding decisions.

At the pan-Canadian event, panelists’ discussions tended to focus on removing provincial barriers to treatment and equitable access to cancer drugs for all Canadians. They also recommended implementing “*equity audits*” so that vulnerable populations are not overlooked.

### Theme 5. Transparency of the decision-making process

Transparency was regarded as foundational to trustworthy governance. Nova Scotia participants stressed that the public needs to understand “*how decisions are made and who is making them*”, while Saskatchewan participants supported “*decision-making that is sustainable, defensible, transparent, objective, accountable, and fair*”.

Participants emphasised that decision-making bodies should represent a range of different perspectives and appropriate expertise, with membership being a mix of citizens, cancer patients, health professionals and policy-makers. There was strong support in the Saskatchewan, Quebec-French, Quebec-English and pan-Canadian panels for excluding the pharmaceutical industry from decision processes due to conflict of interest concerns. However, some Ontario participants supported including industry in these processes so they could be made accountable for their data.

There was some hesitation about the direct involvement of patients in drug funding decisions. For instance, some participants felt patients may be too emotionally tied to decisions under consideration (Nova Scotia, Quebec-French, Quebec-English). This led to some support for recommending that decisions have input from “*patient advocates*” (Quebec-English) and “*patient associations*” (Quebec-French) to avoid placing individual patients under any emotional stress.

Participants strongly supported involving citizens in decision processes, but the support was qualified by a concern that publics might not be adequately informed.

### Theme 6. Pan-Canadian approach to cancer drug funding and coverage decisions

Participants strongly supported the principle that people with similar needs should receive the same care regardless of where in Canada they live, and that fairness should guide a pan-Canadian approach to cancer drug funding. While some participants were sceptical about the ability of Canadian provinces and territories to collaborate on this goal, many were not, and even the sceptics were supportive of the ideal.

Participants in Nova Scotia and at both Quebec panels (English and French) recommended that the same cancer drugs be available in all the provinces and territories, and pan-Canadian panellists recommended a “*mandatory pan-Canadian approach to cancer drug funding decisions*”. At both Quebec panels, participants viewed a Canada-wide drug formulary as a way to mitigate regional differences in treatment access within the province, and all panels viewed it as a way to decrease costs through bulk purchasing. Participants in Saskatchewan considered a common drug formulary primarily as an opportunity to share information and avoid duplication of decision effort across provinces.

## Discussion

Our findings affirm many aspects of current decision-making practices in Canada related to the funding of cancer drugs. Recommendations that drug funding decision processes consider a range of inputs and evidence, that increases in cancer drug spending be justified using clear and consistent principles, and that decision-making processes and their rationales should be transparent and publicly available are consistent with current approaches taken by pan-Canadian health technology assessment (HTA) bodies like CADTH.

Participants wanted drug funding decision processes supported by a range of inputs and evidence, including drug costs, clinical benefit of quality and quantity of life, potential side effects, and incidence rates. Again, CADTH currently considers clinical and economic evidence in its drug assessments, as do Canadian provinces and territories, in their decisions about which drugs to place on their formularies. While CADTH considers input from patient groups in its drug assessment processes, participants expressed some concern about including patients and members of the public in such processes.

Participants wanted further assurances of trustworthy governance of decision-making processes, like avoiding conflicts of interest, committee membership renewal and disclosing reasons for granting compassionate access to drugs not listed on a provincial formulary. These recommendations applied not only to CADTH, but also to provincial and territorial HTA processes to help policy-makers improve how drug funding decisions are made and how their rationales are communicated to the public.

Our findings suggest that new policy options be considered. Participants supported developing processes for re-reviewing data and making disinvestments, ensuring fairness and equity are central principles, and that there should be a pan-Canadian approach to cancer drug funding decisions. While there are currently no formal mechanisms at the pan-Canadian, provincial or territorial levels to either re-review data on effectiveness and cost-effectiveness of currently approved and used cancer drugs or to disinvest in any area of cancer treatment, CADTH’s Strategic Plan for 2018–2021 has focused on HTA management, which includes reassessment and disinvestment of existing drugs and technologies [25]. Participants also recommended that real-world effectiveness and cost-effectiveness be considered in the ongoing re-review of approved drugs in order to achieve better value for money. These recommendations reflect that participants accepted the need for trade-offs and tough funding decisions. Participants also supported replacing an existing drug with a new drug on the basis of cost savings if the new drug has the same safety and effectiveness. Finally, all jurisdictions should give priority to cancer drugs that offer improvements in survival and restore patients’ independence, mental health and general well-being.

Participants recommended that all jurisdictions ensure that people with similar needs receive the same care regardless of where in Canada they live. Because healthcare delivery in Canada, including reimbursement for cancer drugs, is a provincial and territorial responsibility, different provinces and territories have different drug formularies, resulting in disparities of drug coverage across the country. Participants across all panels considered these disparities to be unfair, and called for a pan-Canadian approach to cancer drug funding as a matter of fairness. While some participants were sceptical about the ability of provinces and territories to collaborate on the goal of a common formulary, many felt that a pan-Canadian approach was still an important goal to pursue. Participants made several recommendations related to improving access in rural and remote locales, and strongly supported public funding for oral treatments over intravenous drugs as a means of reducing barriers to treatment access.

There are some limitations to the findings from this project. One limitation relates to the representativeness of the participants. While the participant sample reflected a balance of key demographic criteria across all six panels, with the exception of under-representation in the 18–24 age range, our recruitment goal was not to duplicate population distributions of life experiences but to over-represent the range of diversity in comparison to the general population. Although we did not recruit participants from every province and none were recruited from the territories, the provinces included across all six panels represented approximately 80% of the Canadian population in 2016 [26].

A second limitation relates to the use of decision scenarios, which showed that participants could make specific cost-benefit trade-offs. The scenarios’ structure was intentionally limited in order to force a specific trade-off; however, this also meant that some trade-offs (e.g. involving rare cancers) were not explored in relation to fairness and sustainability, and some decision assumptions (e.g. stage of disease, overall budget) constrained the trade-offs considered.

## Conclusion

The findings from this project provide a set of perspectives on what participants collectively thought made for good, trustworthy decisions about funding for cancer drugs in Canada that are affordable, sustainable and fair. The results demonstrate that informed, deliberative publics accept the need to make trade-offs when budgets are limited. Participants supported making disinvestments based on real-world effectiveness and cost-effectiveness if patients can remain on their current treatment while alternatives are considered. Our approach to public engagement has the strength of producing advice that is informed, considers different social perspectives and reflects collective priorities rather than aggregating individual preferences or highlighting diverse stakeholder perspectives. Policy-makers can use this advice along with other important inputs, including the expertise of clinicians, ethicists, health economists, policy analysts, policy-makers, patients and families.

Canadian policy-makers might want to consider a more sustained public deliberation model, like a public panel or incorporating multiple members of the public into existing committees. While we, as researchers in the fields of public engagement and health policy, have a clear interest in promoting the adoption of robust methods for engaging the public, we believe our approach has demonstrated that civic-minded citizens can effectively contribute to health policy considerations.

The next steps for policy-makers should be to develop mechanisms for (1) re-reviewing effectiveness and cost-effectiveness data for all cancer drugs; (2) making disinvestments in cancer drugs that satisfy requirements relating to grandfathering and compassionate access; (3) ensuring fair and equitable access to cancer drugs for all Canadians; and (4) a pan-Canadian approach to cancer drug funding decisions.

## Additional files

Additional file 1: Citizen brief for pan-Canadian panel. (PDF 3689 kb)

Additional file 2: List of recommendations by province and deliberative topic. (PDF 824 kb)

Additional file 3: List of recommendations from the pan-Canadian panel. (PDF 696 kb)

## Abbreviations

CADTH: The Canadian Agency for Drugs and Technology in Health
HTA: Health technology assessment
