# Supplementary material for: Addressing the affordability of cancer drugs: using deliberative public engagement to inform health policy
Source: Health Res Policy Syst. 2019 Feb 7;17:17. doi: 10.1186/s12961-019-0411-8 (PMC6367823; doi:10.1186/s12961-019-0411-8)
Supplement: Supplementary file 1 — Citizen brief for pan-Canadian panel. (PDF 3689 kb) [file 12961_2019_411_MOESM1_ESM.pdf]

CITIZEN BRIEF

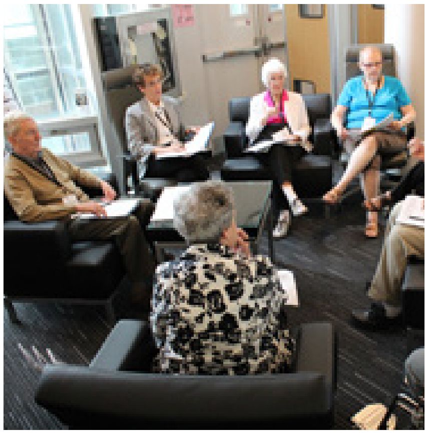

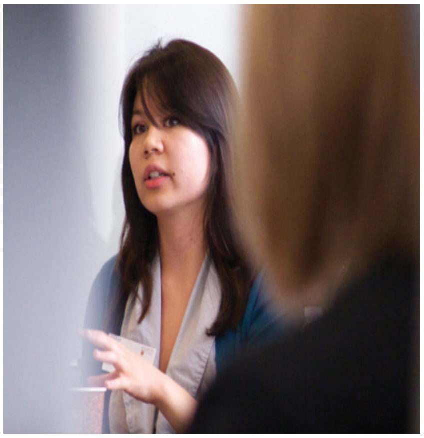

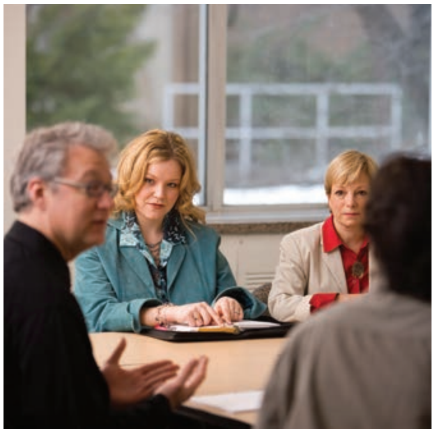

MAKING FAIR AND SUSTAINABLE DECISIONS ABOUT FUNDING FOR CANCER DRUGS IN CANADA

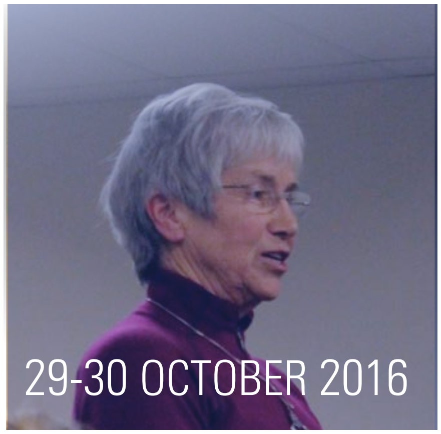

29-30 OCTOBER 2016

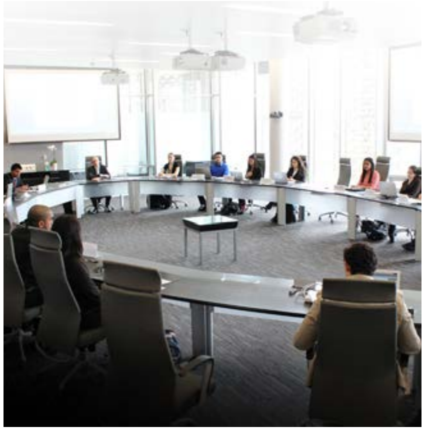

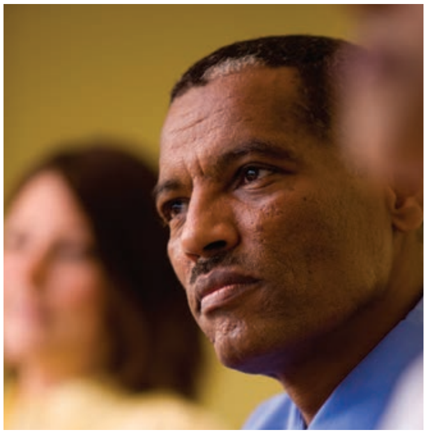

McMaster University

McMaster HEALTH FORUM

ARCC Canadian Centre for Applied Research in Cancer Control

UBC

SFU SIMON FRASER UNIVERSITY ENGAGING THE WORLD

McMaster Health Forum

For concerned citizens and influential thinkers and doers, the McMaster Health Forum strives to be a leading hub for improving health outcomes through collective problem solving. Operating at regional/provincial levels and at national levels, the Forum harnesses information, convenes stakeholders and prepares action-oriented leaders to meet pressing health issues creatively. The Forum acts as an agent of change by empowering stakeholders to set agendas, take well-considered actions and communicate the rationale for actions effectively.

Canadian Centre for Applied Research in Cancer Control (ARCC)

ARCC's mission is to improve cancer control and the delivery of cancer care through interdisciplinary, pan-Canadian leadership in health economics, services, policy and ethics research, education, and knowledge translation. ARCC represents a unique inter-provincial and interdisciplinary initiative based on a collaboration between academics, clinicians and decision-makers from across Canada. The centre is the first of its kind in the world, bringing together some of Canada's leading social science, population health and clinical researchers, and decision-makers.

About citizen panels

A citizen panel is an innovative way to seek public input on high-priority issues. Each of the five panels being convened on the topic of making fair and sustainable decisions about funding for cancer drugs in Canada will bring together 25-30 citizens from all walks of life from the province they are held in. Panel members will share their ideas and experiences on this topic, and learn from research evidence and from the views of others. The discussions of a citizen panel can reveal new understandings about an issue and spark insights about how it should be addressed.

About this brief

This brief was produced by the McMaster Health Forum and ARCC to serve as the basis for discussions by the citizen panel on making fair and sustainable decisions about funding for cancer drugs in Canada. This brief provides information on this topic, including what is known about the underlying problem, possible elements of an approach to address the problem and potential barriers and 'windows of opportunity' for implementation.

This brief does not contain recommendations, which would have required the authors to make judgments based on their personal values and preferences.

Table of Contents

Key Messages ... 1

Questions for the citizen panel ... 2

The context: Why is making fair and sustainable decisions about funding for cancer drugs a priority in Canada? ... 5

Trade-offs have to be made ... 11

Making trade-offs is difficult without insights from citizens ... 12

The problem: Why is making fair and sustainable decisions about cancer-drug funding necessary but challenging? ... 14

The burden of cancer is high and continues to grow ... 15

The amount spent on cancer drugs is increasing ... 16

The way some cancer drugs are paid for puts strain on patients and their families, as well as the health system ... 18

Addressing the situation requires making fair but difficult decisions ... 19

Decisions about cancer-drug funding affect some groups more than others ... 20

Elements of an approach to address the problem ... 21

Element 1 – Getting evidence to the decision-making table ... 22

Element 2 – Including relevant perspectives in the decision-making process ... 24

Element 3 – Acting on decisions to achieve impact ... 26

Implementation considerations ... 28

Acknowledgments ... 31

References ... 32

Key Messages

What's the problem?

Several factors contribute to the challenge of making fair and sustainable decisions about funding for cancer drugs in Canada:

- the burden of cancer is high and continues to grow;
- the amount spent on cancer drugs is increasing;
- the way some cancer drugs are paid for puts strain on patients and their families, as well as the health system;
- addressing the situation requires making fair but difficult decisions; and
- decisions about cancer-drug funding affect some groups more than others.

What are elements of a potentially comprehensive approach for addressing the problem?

- **Element 1:** Getting evidence to the decision-making table
    - *Evidence to consider*: A number of things might be considered when making funding decisions about healthcare interventions, which include (but are not necessarily limited to): 1) burden of disease; 2) potential benefits and harms; 3) cost-effectiveness; 4) availability of alternative interventions; 5) values and preferences of patients and citizens; 6) equity (i.e., fairness); 7) acceptability; and 8) feasibility.
    - *Overarching question to consider*: What information is important to support decision-making about whether to fund new cancer drugs or change the funding provided for existing drugs?
- **Element 2:** Including relevant perspectives in the decision-making process
    - *Evidence to consider*: Effective decision-making groups often include a broad range of stakeholders, including patient and citizens, individuals with content and technical expertise, an effective leader to facilitate collaboration, and training and support for members who may not be familiar with all of the concepts discussed.
    - *Overarching question to consider*: What would make decision-making processes for cancer-drug funding trustworthy?
- **Element 3:** Acting on decisions to achieve impact
    - *Evidence to consider*: Greater drug coverage may reduce the use of other healthcare services, shifting costs to the patient may reduce government drug costs but may also lead to worse health outcomes and more use of other services, and prior approval for the use of some drugs can reduce costs and improve their use.
    - *Overarching question to consider*: What would make approaches to acting on decisions about funding cancer drugs trustworthy?

What implementation considerations need to be kept in mind?

- Challenges to implementing these elements might include: 1) provincial and territorial health-system leaders may have different readiness for change and collaboration; 2) groups (e.g., patient groups, disease-specific groups or pharmaceutical industry) mobilizing to support approval and funding for certain drugs and/or to oppose decisions to not fund certain drugs; and 3) public concern about why funding decisions for cancer drugs are treated differently tha other drugs.
- Windows of opportunity for implementing these elements might include: 1) a growing desire within the health sector for pan-Canadian leadership and collaboration; 2) difficult economic times forcing the creation of innovative approaches; and 3) public interest in having a voice in decisions that affect them.

Questions for the citizen panel

>> We want to hear your views about the problem, three elements of a potentially comprehensive approach to addressing it, and how we can address barriers to moving forward.

This brief was prepared to stimulate the discussion during the citizen panel. The views and experiences of citizens can make a significant contribution to finding the best ways to meet their needs. More specifically, the panel will provide an opportunity to explore the questions outlined at the beginning of each section. Although we will look for common ground during these discussions, the goal of the panel is not to reach consensus, but to gather a range of perspectives, including areas of agreement and persistent disagreement, on the topic of making fair and sustainable decisions about funding for cancer drugs.

We provide in Box 1 an overarching question for you to consider, as well as some more specific questions to think about when reading the brief. We provide additional questions for you to consider in the section about the elements of a potentially comprehensive approach to addressing the problem.

Box 1: Questions to consider for your deliberations

Overarching question to consider

- How can funding decisions about cancer drugs be made in a way that is fair and sustainable?

Additional questions (please also see those provided as part of each element)

- How should we approach making decisions to fund new cancer drugs?
- How should we approach making decisions to stop or limit funding for some cancer drugs now being used?
- What would make you trust the decisions that are made about the funding of cancer drugs, or for actions taken to implement these decisions?
- Should drug funding decisions for cancer be treated differently than funding decisions for other drugs? Why?

Box 2: Glossary

Terms related to cancer drugs

Chemotherapy

"…a drug treatment that uses powerful chemicals to kill fast-growing cells in your body. Chemotherapy is most often used to treat cancer, since cancer cells grow and multiply much more quickly than most cells in the body. Many different chemotherapy drugs are available. Chemotherapy drugs can be used alone or in combination to treat a wide variety of cancers. Though chemotherapy is an effective way to treat many types of cancer, chemotherapy treatment also carries a risk of side effects. Some chemotherapy side effects are mild and treatable, while others can cause serious complications."(5)

Biological therapy and immunotherapy

"…a type of treatment that uses the body's immune system to kill cancer cells. Biological therapy for cancer is used in the treatment of many types of cancer to prevent or slow tumor growth and to prevent the spread of cancer."(9)

Terms related to evidence

Cost effectiveness

A measure of the health gains achieved for money spent, which is often measured through what's called *quality adjusted life years*, and which is determined through a technical assessment that includes information from clinical trials and studies of patient preferences.

Opportunity cost

A potential benefit that has to be given up to achieve something else.

Quality of life

A broad concept often referring to assessments of positive and negative aspects of life.(12)

Clinical trials

The most common source of medical evidence about a drug. They are used to test when a drug is safe to use and if it produces health benefits. They also check how often serious adverse events happen, like death, hospitalization and disability.(4)

Systematic reviews

A synthesis of results from all the studies addressing a specific topic.

Box 3: Glossary (continued)

Terms related to making decisions

Advisory committee

A committee made up of individuals with different expertise and perspectives on the drug under consideration, including patients who have experienced the health condition for which the drug is intended. Advisory committees provide advice – in the form of recommendations – to decision-makers.(4)

Priority setting

The process of making decisions about what programs, drugs and technologies to support when there aren't enough resources to support them all.(4)

Trade-offs

"Priority setting involves trade-offs. A trade-off means that the resources – e.g., people, time, money, hospital beds, organs for transplant – used in one place can't be used in another place at the same time."(4)

Terms related to acting on decisions

Drug approval process

Health Canada approves drugs (and other products) for marketing and sale in Canada by assessing the safety, benefits and quality of a drug based on evidence submitted by the company seeking approval.(11)

Public drug formulary

The list of drugs and drug products that are paid for by a provincial government drug plan. Not all drugs approved for use by Health Canada are listed on a given province's public drug formulary.(4)

Disinvestment & de-listing

Setting priorities may include shifting funding from one drug to another. Disinvestment refers to when the money being spent on one drug is withdrawn (i.e., disinvested) and then used to pay for another, more effective drug. De-listing is a process where the money spent on one drugs is withdrawn (i.e., it is removed from the public drug formulary).

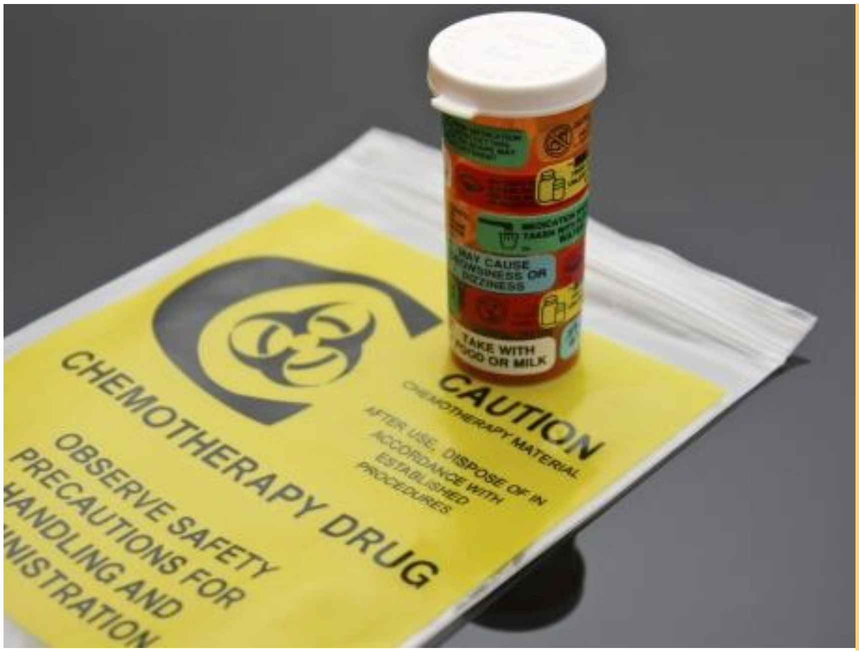

Making decisions about what drugs to cover is difficult without insights from citizens

The context: Why is making fair and sustainable decisions about funding for cancer drugs a priority in Canada?

Provincial governments are concerned about making drug funding decisions in a fair and sustainable way. This includes priorities about which cancer drugs should be funded. These decisions are difficult, especially when a high price tag for a drug needs to be considered against what may often be small increases in health benefits. These decisions are even harder when other less expensive drugs may be available that may provide only slightly less health benefits.

Decisions about these types of trade-offs among potential benefits, harms and costs of a drug involve large investments of public resources. Since these investments are often made at the expense of other priorities, it is important for policymakers to have input from a wide range of people affected by the issue, including input in the form of the values and preferences of citizens. As background to the discussion, we describe in Table 1 the current processes used to make these trade-offs across Canada, with the exception of Quebec where assessments and

recommendations are made by the Institut national d'excellence en santé et en services sociaux (INESSS). We also provide an overview of government drug coverage in Box 4.

Table 1. Summary of cancer drug approval and funding processes (4;13)

Steps	Description
**Step 1 – Authorize for sale (by Health Canada)**	• A pharmaceutical manufacturer seeking to sell a new drug in Canada submits an application to Health Canada for review.<br>• Health Canada assesses the scientific information on the drug's safety, clinical efficacy and the quality of its manufacturing process. Clinical efficacy is how well the drug works to prevent or control disease and improve health.<br>• Health Canada's approval of a drug for sale in Canada does not necessarily mean that government drug plans will fund it.
**Step 2 – Recommend to fund or not (through the Canadian Agency for Drugs and Technologies in Health's (CADTH) pan-Canadian Oncology Drug Review (pCODR))**	• CADTH's expert advisory committees review the medical research evidence (i.e., usually clinical trials) and costs to determine if the drug works better than the usual treatment, and if the cost is reasonable.<br>• Taking patient and citizen values and preferences into account, CADTH then makes one of three types of funding recommendations to the provinces: 'Recommend,' 'Consider with conditions,' or 'Do not recommend.'
**Step 3 – Decide to fund (by provincial ministries of health and cancer authorities)**	• Provincial ministries of health and cancer authorities use the recommendations to make their own decisions, and may consider input from their own expert committees and base decisions on other considerations (e.g., effect on health services and its budget). CADTH recommendations are not binding on government drug programs.<br>• After receiving funding approval in a province or territory, a drug will typically be added to the public drug formulary.

Box 4a: Overview of government cancer drug coverage in Saskatchewan

There is no national government-funded drug plan in Canada, and therefore the nature and extent of government drug plans are different for each province. We provide a description of cancer drug coverage in Saskatchewan below.

- The Saskatchewan Cancer Agency pays the full cost of all approved cancer drugs, including injectable and oral drugs and those taken in hospital and at home, for residents with a valid health card who are registered at the Cancer Agency.(6)
- For those who are clinically unable to receive what is considered to be the standard treatment approach, the Saskatchewan Cancer Agency will consider funding a drug on an exceptional case-by-case basis. To access such funding, a request from a physician is reviewed by the Agency's Pharmacy and Therapeutics Committee who approve or deny a request based on their review of the clinical merits of the request, budgetary impact, and whether the drug is approved by Health Canada for the purpose it has been requested for.
- Public coverage for other prescription drugs is provided through several programs, including:
    - Children's Drug Plan, which requires a $20 payment for each prescription for children aged 14 and younger;
    - Emergency Assistance for Prescription Drugs, which provides a limited supply of approved prescription drugs at a reduced cost (determined based on an individual's ability to pay) for those who are in need of immediate treatment, but unable to pay for their share of the cost;
    - Senior's Drug Plan, which requires those aged 65 years and older to pay a maximum of $20 per prescription; and
    - Special Support Program, an income-tested program for those with high drug costs in relation to their income, which requires those who are eligible to pay a deductible and/or a co-payment for their prescriptions in each calendar year.(10)

Box 4b: Overview of government cancer drug coverage in Ontario

There is no national government drug plan in Canada, and therefore the nature and extent of government drug plans is different for each province. We provide a description of cancer drug coverage in Ontario below.(1)

- Approved cancer drugs given to patients in outpatient clinics of hospitals and cancer centres are paid for those with a valid health card through programs run by Cancer Care Ontario and hospital/cancer centre budgets (with funding from government).
- Funding for injectable and oral drugs not typically covered by public plans can be accessed on a case-by-case basis for cancer patients with rare clinical circumstances.
- Public coverage is not provided for cancer drugs taken at home, unless the patient is eligible for coverage through the Ontario Drug Benefits program that is offered to seniors, those on social assistance, and those living in special care or long-term care homes. Individuals receiving coverage through this program may be required to pay a deductible (an amount an individual has to pay themselves before being eligible to receive benefits) and a co-payment (a fee for each prescription filled).
- Families with high drug costs in relation to their income can also access the Trillium Drug Program, which requires patients to pay a deductible and a co-payment for each prescription filled.
- Those with no or partial coverage through these programs need to pay for drugs through private insurance or out-of-pocket.

Box 4c: Overview of government cancer drug coverage in Quebec

There is no country-wide government-funded drug plan in Canada, and therefore the nature and extent of government drug plans are different for each province. We provide a description of cancer drug coverage in Quebec below.(7;8)

- Cancer drugs provided as part of in-patient treatment at cancer centres or at community hospitals are fully paid for, with each hospital having a list of medications that can be used (but these lists can vary between hospitals).
- Cancer drugs taken at home are not paid for by hospitals, but may be paid for by the Public Prescription Drug Insurance Plan or private insurance plans.
- All residents of Quebec are required to have prescription drug coverage, either through the Public Prescription Drug Insurance Plan or private insurance.
- The Public Prescription Drug Insurance Plan is administered by the Régie de l'assurance maladie du Québec (RAMQ) and is intended for persons who are not eligible for a private group insurance plan that provides coverage for prescription drugs, for people aged 65 or over, and for recipients of last-resort financial assistance.
- Those insured under the public plan are required to make a monthly contribution towards their drugs, which varies for different groups:
  - people aged 18 to 64 not eligible for a private plan or people aged 65 and over who do not receive the guaranteed income supplement pay a maximum monthly contribution of $85.75 ($1,029 maximum yearly contribution); and
  - people aged 65 and over who receive 1% to 93% of the Guaranteed Income Supplement pay a maximum monthly contribution of $51.83 ($622 maximum annual contribution).
  - No contribution is required for three groups:
    1) holders of a claim slip issued by the Ministère de l'emploi et de la solidarité sociale (for recipients of last-resort financial assistance, which give access to certain drugs and healthcare services such as eye exams and dental care);
    2) people over the age of 65 or those receiving 94% to 100% of the Guaranteed Income Supplement; and
    3) children of those insured under the public plan (if they are under age 18 or if they are between ages 18 and 25, are spouseless, live with their parents, and are full-time students in an educational institution at the secondary, college or university).
- People receiving public coverage may have to pay more for certain drugs covered under the List of Medications, with the list being determined by the Ministère de la santé et des services sociaux, in consultation with the Institut national d'excellence en santé et en services sociaux.
- For treatment of a serious condition, a drug not listed on the List of Medications may be covered on an exceptional basis. A patient's doctor must send a payment authorization request to the RAMQ. If authorization is denied, the patient or their doctor can submit a written request to review the decision within six months. If the patient is not satisfied with the decision following the review, they can appeal to the Tribunal administratif du Québec within 60 days.

Box 4d: Overview of government cancer drug coverage in Nova Scotia

There is no national government-funded drug plan in Canada, and therefore the nature and extent of government drug plans are different for each province. We provide a description of cancer drug coverage in Nova Scotia below.(3)

- The provincial government pays for approved cancer drugs given to patients with a valid health card in hospitals, cancer centres and outpatient clinics.
- Public coverage is not provided for cancer drugs taken at home unless the patient is eligible for one or more of the provincial government-funded drug plans outlined below.
  - *Drug Assistance for Cancer Patients* is a provincial drug plan available to low-income residents (those with an annual family income of no more than $15,720) under the age of 65 with no other drug coverage, except for Family Pharmacare (see immediately below). Once approved, the plan pays for all cancer-related drugs and supplies.
  - The *Nova Scotia Family Pharmacare Program* is available to protect families if they have no drug coverage or very high prescription drug costs, and is available to all residents with a valid health card. Families are required to pay an annual deductible and co-payment, both of which are based on income and family size. For example, a family with two children and an annual income of $20,000 would be required to pay a deductible of $140 each year, as well as a co-payment of 20% per prescription up to $700 per year. For a family with two children and an annual income of $100,000 the deductible would be $17,390 and the annual co-payment limit would be $14,100. Once the family pays both the deductible and annual co-payment amounts, all drug costs are covered.
  - The *Seniors Pharmacare Program* pays for drugs and devices on the Nova Scotia Formulary. Single seniors earning less than $22,986 are not required to pay a… premium, while others pay up to $424 per year. Couples with a combined income between $26,817 and $40,000 each pay a reduced premium of less than $424 per year.
  - The *Palliative Care Drug Program* covers all drugs for those who have been assessed by a palliative-care team to be in the end stage of a terminal illness (defined as being in the last six months of life), and who wish to receive end-of-life care at home or in a supportive-living residence.
  - The Department of Community Services' *Pharmacare Benefits Program* provides drug coverage to *Income Assistance* clients, *Disability Support Program* clients, children in the care of child welfare, or *Low Income Pharmacare for Children* clients. Eligible beneficiaries receive help with the cost of certain prescribed drugs, devices and related services dispensed by pharmacists.

Box 4e: Overview of government cancer drug coverage in British Columbia (BC)

- There is no national government drug plan in Canada, and therefore the nature and extent of government drug plans are different for each province. We provide a description of cancer treatment and supportive care drug coverage in BC below.
- Approved cancer drugs given to BC patients at one of the BC Cancer Agency's six regional cancer centres or in community hospitals are paid for primarily by the BC government, which funds cancer medications through the BC Cancer Agency (BCCA).
- The BCCA has a Compassionate Access Program to support access to evidence-based cancer treatments that are restricted in funding or indicated in exceptional clinical circumstances. Applications are evaluated according to the supporting evidence and the cost impact for the BCCA.
- Cancer patients may receive ambulatory treatment for funded treatment protocols at regional cancer centres or at Community Oncology Network (CON) clinics within community hospitals in the province. These treatments are delivered at no cost to BC residents.
- Cancer drugs taken at home that are funded by the BCCA are dispensed by and paid for through regional cancer centre pharmacies or CON clinic pharmacies. These take-home drugs can include oral chemotherapy drugs, oral hormonal medications, injectable hormonal agents that are taken to personal doctors' offices for administration, and bone metabolism regulators.
- Cancer drugs taken at home that are not funded by BCCA are purchased by patients in retail pharmacies or through patient assistance programs. These can also include oral chemotherapy drugs, oral or injectable hormonal agents, or bone metabolism regulators being used outside of funded BCCA indications.
- Supportive care medications, such as anti-nausea drugs and pain medications, are not funded through the BCCA. BC Pharmacare administers a number of benefit plans for BC residents for coverage for supportive care medications. For instance, Fair Pharmacare ensures that the BC Pharmacare annual deductible is set at a level based on household income.
- Individuals may also have private insurance programs to assist with supportive care medication costs or for unfunded cancer treatment medications.
- Some patients might choose to receive cancer treatment drugs outside of the BCCA's funded indications. These can be either oral or intravenous drugs. In this scenario, the patient pays for the oral treatments at a retail pharmacy and self-administers at home. For unfunded intravenous treatments, some patients may choose to be treated at private infusion clinics throughout the province.

Trade-offs have to be made

Cancer is the leading cause of death in Canada (14;15) and poses a significant challenge to health systems in Canada given rising costs of drugs.

The amount of money spent on cancer drugs is increasing in part because of growing numbers of new cancer drugs and rising prices for them. When there are not enough resources to fund everything needed it can be challenging to make both fair and sustainable decisions about which drugs to fund. Trade-offs have to be made in light of the potential health benefits and possible harms of a drug, how many patients stand to benefit from the drug, and its costs, among other factors. Trade-offs also have to be made with an awareness of the already overstretched health system budgets, and difficult economic times in the country.

Arguably, decisions about funding potentially valuable but high-cost cancer drugs need to consider what those resources can't be used for. For example, providing funding for one high-cost drug for some may mean not being able to cover other services, technologies or drugs in other parts of the health system.

Making trade-offs is difficult without insights from citizens

Addressing these challenges means thinking about whether and how to make changes to the way we make decisions as outlined (below) in Figure 1. This means ensuring we are:

1. getting evidence to the decision-making tables in provincial ministries of health;
2. including relevant perspectives in the decision-making process; and
3. acting on decisions to achieve impact.

Figure 1: Example of a general approach to policy decision-making

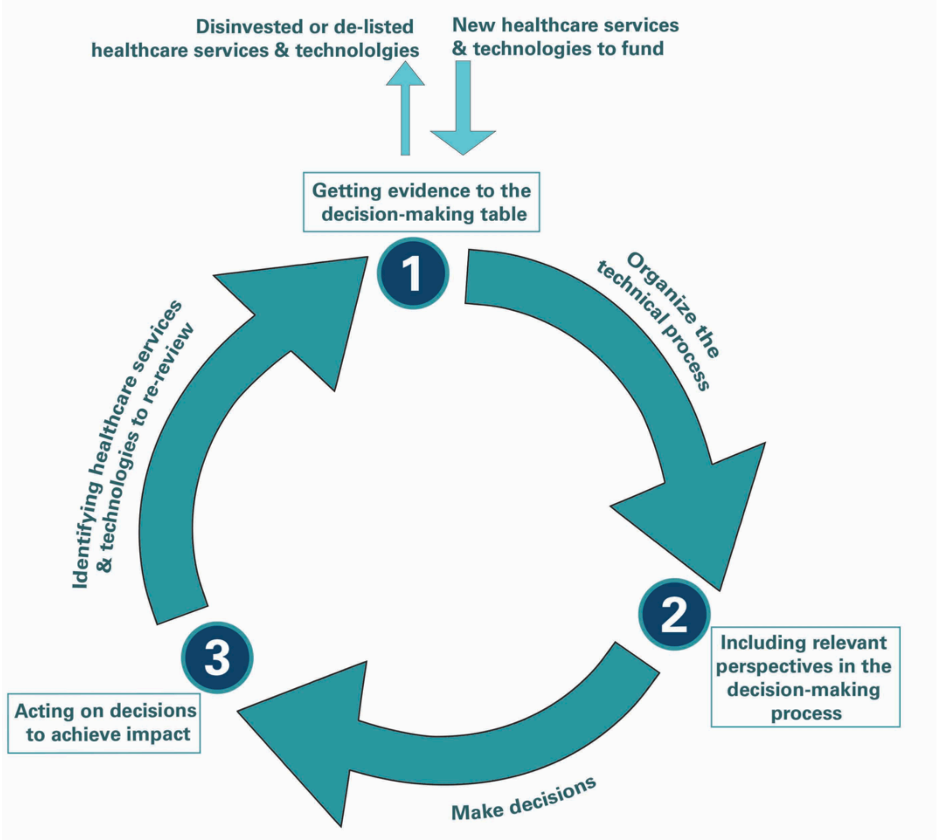

This is a general overview of a decision-making process, and in reality there are many challenges across each of the steps. For example, technical processes often have to work with evidence that does not provide a clear picture of the trade-offs involved. Also, decision-making processes may engage a broad range of groups (including citizens), but the reasons behind the final decision may not be made clear to the public. Lastly, decision-making processes often do not re-review currently funded services to identify what should no longer be covered or where coverage should be provided only for some people. We discuss specific challenges related to decision-making about cancer-drug funding in the problem section later in the brief.

Insights from citizens through public engagement can help to inform how we address these challenges to make fair and sustainable decisions about cancer-drug funding in provincial health systems. A public deliberation was held in Vancouver, British Columbia in 2014 about setting funding priorities for cancer drugs, but given that cancer drug coverage varies by province it is important to conduct public deliberations in other provinces as well. Such deliberations can identify similarities and differences in citizens' values and preferences about how to make fair and sustainable decisions about cancer-drug funding.

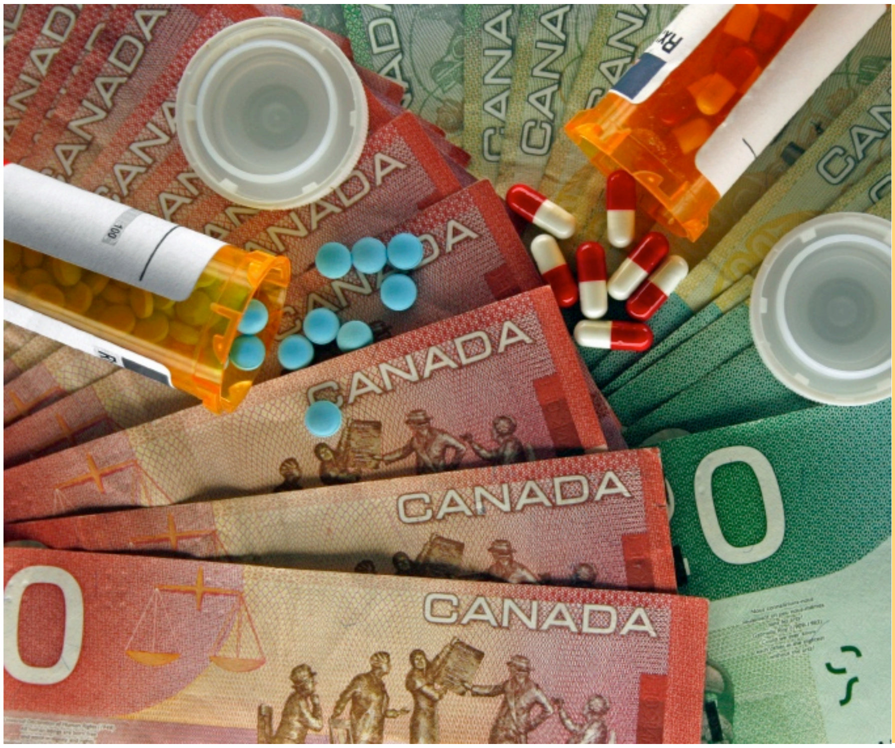

As the burden of cancer continues to grow, so does the number of and prices for new cancer drugs

The problem: Why is making fair and sustainable decisions about cancer-drug funding necessary but challenging?

Several factors contribute to the challenge of making fair and sustainable decisions about cancer drugs in Canada:

- the burden of cancer is high and continues to grow;
- the amount spent on cancer drugs is increasing;
- the way some cancer drugs are paid for puts strain on patients and their families, as well as the health system;
- addressing the situation requires making fair but difficult decisions; and
- decisions about cancer-drug funding affect some groups more than others.

The burden of cancer is high and continues to grow

Cancer is the leading cause of death in Canada.(15) However, it is important to note that death is not the only measure of the importance of a disease. For example, other illnesses such as cardiovascular disease, diabetes, mental health issues and osteoarthritis are more frequent than cancer,(16) and may have longer-term impacts on quality of life.

Figure 2 portrays the burden of cancer in Canada. The burden of cancer is growing as both the number of new cancer cases diagnosed each year is rising, and the total number of people living with cancer is increasing.(14) More than half of new cancer cases (51%) will be lung, breast, colorectal and prostate.

The Canadian Cancer Society estimates that by 2028-2032, the average annual number of new cancer cases will increase 79% compared to 2003-2007. The main cause of the increase is an aging and a growing population.

The burden of cancer on the health system is significant. Cancer is one of the 10 costliest illnesses or injuries in Canada.(17) Given that the number of new cancer cases continues to grow each year, these costs are on the rise. For example, a recent analysis found that over a 10-year period (1997-2007) initial cancer treatments increased by 50% for prostate and lung cancer, doubled for breast and colorectal cancers, and tripled for melanoma.(18)

Figure 2: Most recent summary of burden of cancer in Canada (figure adapted from the Canadian Cancer Society) (14)

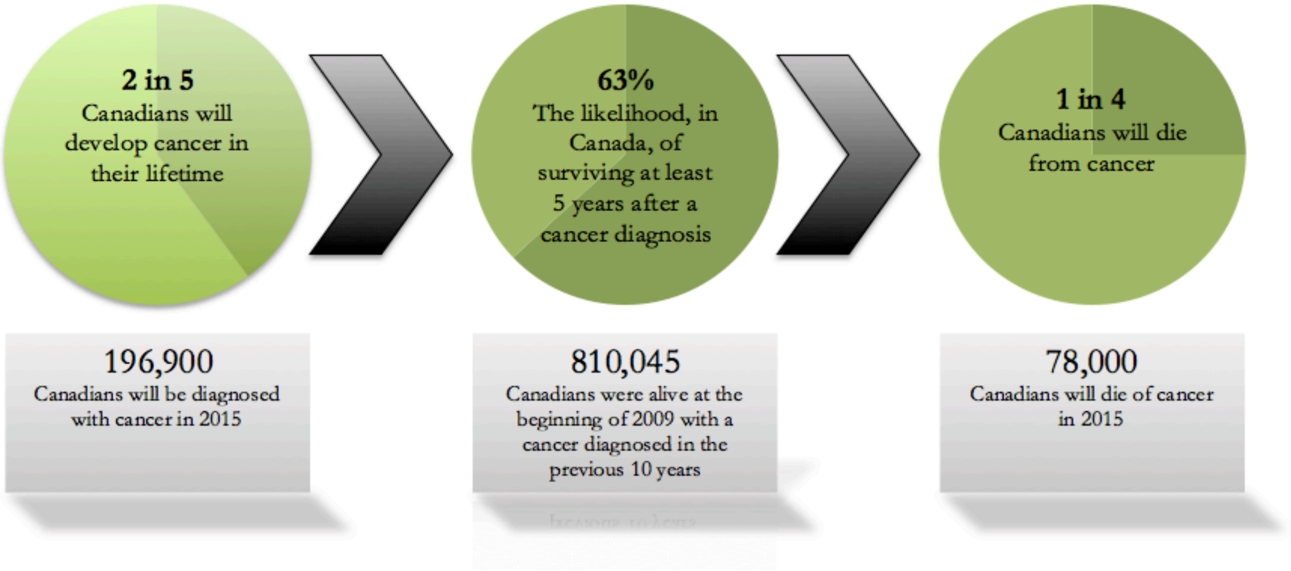

The amount spent on cancer drugs is increasing

There are more drugs available to treat cancer than ever before, which also means that cancer drugs are a big contributor to the health-system costs across the country. In considering these costs, much attention is often paid to drugs with very high prices. However, some less expensive drugs that are used very frequently can also end up having a large impact on the overall budget. What's important is to ensure that fair access is provided to valuable drugs, and in a way that is affordable for both patients and their families being treated within the health system, and the Canadian public that is paying for the health system.

In 2015 overall drug expenditure was estimated to be $959 per person, which was a 0.7% increase from the previous year,(19) and puts Canada as one of the largest per capita spenders on drugs in the world (only less than the United States, Japan and Greece).(20) This accounted for the second-largest share of health spending in Canada (15.7%), second only to hospital spending (29.5%), and more than spending on physicians.(19) This expenditure also does not include the amount spent on drugs that are provided in hospitals, and a recent report indicates that in 2009, approximately $2.4 billion was spent on drugs provided in hospitals.(21;22)

Of the amount spent on all drugs in hospitals, one-third ($800 million) was spent on cancer drugs.(21;22) The price of drugs to treat cancer is also on the rise year after year. In some provinces expenditure on cancer drugs has more than doubled in the last 10 years. For example, between 2005 and 2015 the amount of money spent by the Ontario government on intravenous cancer drugs tripled from $112 million to $332 million per year.(23)

Another important consideration is that new cancer drugs are always being developed and often come with a large price tag. As an example of this, we have provided in Box 5 below an excerpt from a recent media article about the high costs of a new drug.

Box 5: Example of high cancer drug costs (2)

New cancer drugs raise hope for patients, but carry steep costs

The skyrocketing price of new cancer therapies — some of which prolong life by only a few months — is leading some to question whether the cost is sustainable.

Examples of high drug costs faced by patients and their families (as cited in the article)

- Between 2005 and 2015 the amount of money spent by the Ontario government on intravenous cancer drugs tripled from $112 million to $322 million per year.
- 11 of the 12 new cancer drugs approved by the U.S. Food and Drug Administration in 2012 cost over US$100,000 per patient per year.
  - Only three of those drugs increased patient survival rates.
  - Two of three increased survival by less than two months.
- A suggested treatment for late-stage melanoma combines two high-cost drugs and would, for the average patient, cost $415,000 per patient.

Case of a 60-year old man who has been in treatment for late-stage chronic lymphocytic leukemia, and who has been told that he is out of treatment options and in the last stages of his life (as cited in the article)

- A clinical trial offers the opportunity to test a new drug called Ibrutinib in conjunction with two types… of chemotherapy.
- As a result of the treatment, he is now alive three years later.
- He currently takes three capsules of the drug each day, and each capsule costs about $100.
- This amounts to nearly $110,000 per year and he has to take the drug for the rest of his life, or until a time when the drug is no longer effective.
- The drug has now been approved for use by Health Canada.

The way some cancer drugs are paid for puts strain on patients and their families, as well as the health system

Provincial governments in Canada have historically paid for both hospital-based and physician-provided care. Because chemotherapy has typically been provided intravenously in hospital, it has been paid for by provincial governments. However, as more cancer care is delivered in outpatient settings (including oral chemotherapy), patients and their families are increasingly responsible for the costs associated with them. For example, in 2014, 6.4 billion (22.2%) of all drug costs (not just cancer drugs) were paid for directly by patients and their families. For the remaining costs, multiple payers are involved with $12.1 billion (42%) of prescribed drugs financed by the public sector and $10.3 billion (35.8%) financed through private insurance.(22)

However, these numbers don't tell the whole story as the extent of responsibility for the cost of cancer care that is placed on the patient differs across provinces. Those living in western Canada, including Saskatchewan, have both intravenous and oral chemotherapy paid for by government regardless of where it is provided. Other provinces like Ontario and the eastern provinces provide coverage for cancer treatment provided in hospitals, but only provide coverage to some patient groups for treatment delivered outside of hospital and cancer centres.

For example, if an individual patient were to undergo a one-year course of treatment with an oral cancer medication that costs $6,000 per month, and her net income was $85,000 per year and she had no private insurance, she could be facing out-of-pocket costs ranging from $1,006 in Quebec and $3,400 in Ontario, to as high as $23,000 in Nova Scotia.(24)

Addressing the situation requires making fair but difficult decisions

It is always difficult to make decisions about whether or not to fund a specific drug, but there are particular challenges with cancer-drug funding that make it even more difficult. One challenge is the need to balance patients' values and preferences with the often uncertain trade-offs among patients' capacity to benefit (e.g., whether the cancer is curable or not, and the likelihood of the drug being able to lengthen life and improve quality of life), potential harms and costs.

For example, high-cost cancer drugs may offer limited or uncertain benefits in situations when there is no alternative treatment available or it is the last-hope treatment. Given finite resources, such decisions about cancer drugs with high price tags also have implications in other parts of the health system, and in other areas where government action is needed.

There are several additional challenges related to bringing evidence to the table, making decisions, and acting on decisions. For bringing evidence to the table, technical processes can only be as good as the studies available at the time decisions need to be made. Also, technical processes have typically been designed to be good at adding drugs to the health system, but not removing them from it.

Turning to making decisions, some may see an obligation for provincial drug formularies to continue funding a drug for those currently using it, even after a decision to constrain its use or de-list it. Although, it could be the case that when a good substitute for an expensive drug arises, those on the more expensive drug switch to the cheaper one, or the more expensive drug is used only in specific situations.

Turning finally to acting on decisions, provincial drug formularies can sometimes be circumvented through other processes that provide compassionate access to drugs and treatments. This can happen in situations where elected officials are confronted with intense public pressure to reverse a decision to not fund a drug for one or more individuals.

Decisions about cancer-drug funding affect some groups more than others

The rising cost of cancer drugs, and decisions about cancer-drug funding, affect some individuals more than others. Those with lower incomes will feel the impact of out-of-pocket drug costs more than those with higher incomes. This results in some low-income individuals not following cancer treatment plans because they can't afford their cancer drugs.(25)

Individuals without private health insurance may also feel the impact of high drug costs more than others. These individuals are typically not employed, or are employed seasonally, part-time or self-employed, and as a result may already be economically disadvantaged.(26) Even those who do have private health insurance may face high out-of-pocket costs given more than 75% of private health plans require patients to make co-payments of 20% or more, and many have lifetime or annual limits on drug coverage.(27)

As individuals move through their cancer journey, their personal situations – and hence the impact of public drug funding decisions – may also change. For example, a high-cost drug may be the only option for those who:

- have tried first-line treatments without success;
- may require more intensive and longer term treatments if a cancer recurs; and
- will face difficulty paying for their cancer drugs if they are not yet eligible to return to full government benefits (e.g., employment insurance) if they have not been back to work for a sufficient number of hours.(26)

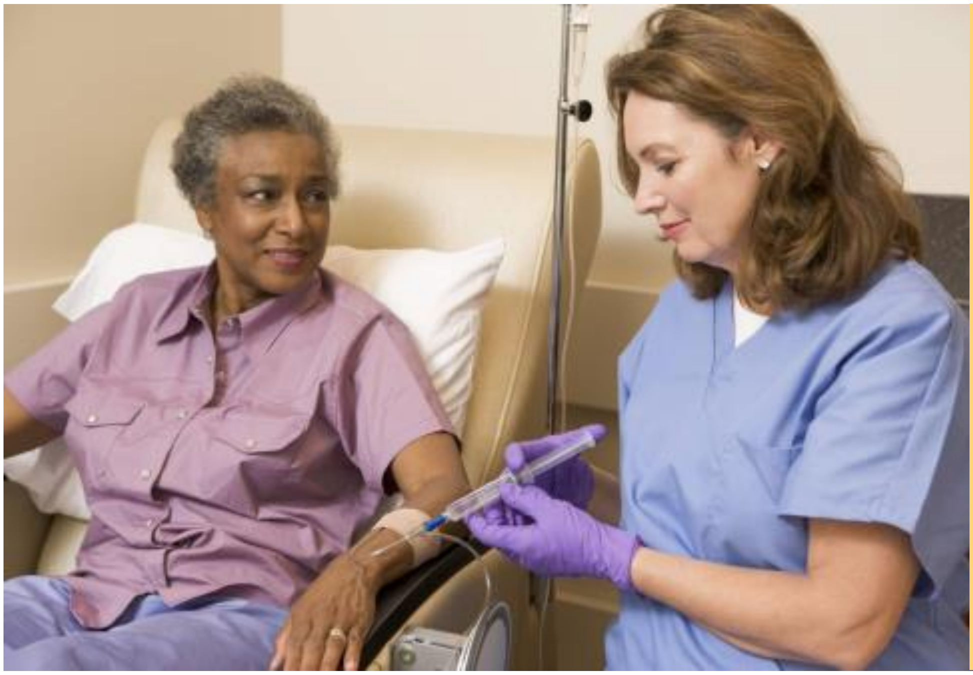

We have selected three elements of an approach to address the problem for which we are seeking public input.

Elements of an approach to address the problem

>> To promote discussion about the pros and cons of potential solutions, we have selected three elements of an approach to making fair and sustainable decisions about funding for cancer drugs in Canada

Many approaches could be selected as a starting point for discussion. We have selected the following three elements of an approach for which we are seeking public input:

1. getting evidence to the decision-making table;
2. including relevant perspectives in the decision-making process; and
3. acting on decisions to achieve impact.

These elements should not be considered separately. Instead, each should be considered as contributing to a potentially comprehensive approach to addressing the problem. New elements could also emerge during the discussions.

Element 1 – Getting evidence to the decision-making table

Overview
This element is focused on the types of information that are important for making decisions about cancer drugs. Evidence and questions to consider during your deliberations are provided below.

Evidence to consider
A number of things might be considered when making funding decisions about healthcare interventions. These include (but are not necessarily limited to):

- burden of disease;
- potential benefits and harms;
- cost-effectiveness;
- availability of alternative interventions;
- values and preferences of patients and citizens;
- equity (i.e., fairness);
- acceptability; and
- feasibility.(28)

It is particularly important that Canadians should have a voice in decisions about the health care that they receive now and in the future, and this requires careful consideration of citizens' values and preferences.

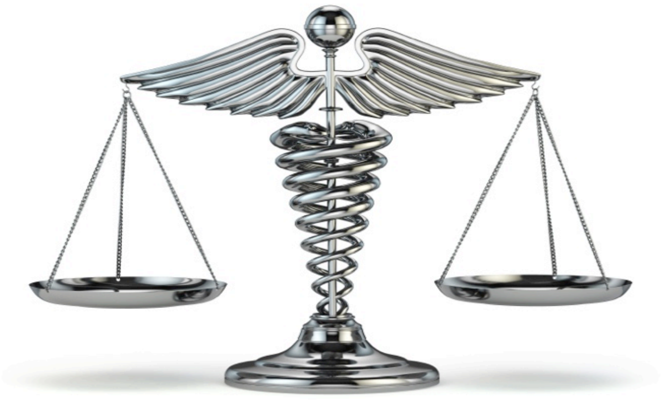

Questions to consider

Overarching questions to consider

- What information is important to support decision-making about whether to fund new cancer drugs or change the funding provided for existing drugs?

Additional questions to consider

- When trade-offs have to be made in decisions about whether to fund new cancer drugs or discontinue funding for existing drugs, how much weight should be given to:
  - potential benefits (e.g, whether the drug can lengthen life or help improve quality of life);
  - potential harms that could happen as a result of taking the drug;
  - costs that have to be paid by public and private drug coverage or by patients and their families;
  - availability of alternative treatments;
  - citizens' values and preferences (e.g., as identified through as citizen panels or other mechanisms); and
  - equity (i.e., fairness)?
- What if a drug is not very expensive, but not very effective?
- Should the severity of a health condition (e.g., whether it is curable or not) or a patient's age matter in decisions about funding cancer drugs?

Element 2 – Including relevant perspectives in the decision-making process

Overview

The focus of this element is on identifying what citizens think would make decision-making processes trustworthy. Evidence and questions to consider during your deliberations are provided below.

Evidence to consider

A review focused on group composition and group processes as part of efforts to improve the use of evidence in the development of clinical guidelines found that:

- the best groups include a broad group of stakeholders (e.g. patients and their families, health professionals, managers and policymakers), individuals with content and technical/methodological expertise, and an effective leader to facilitate collaboration and contributions from all group members; and
- training and support should be made available to members of the group who may not be familiar with all of the methods and processes used to develop recommendations.(29)

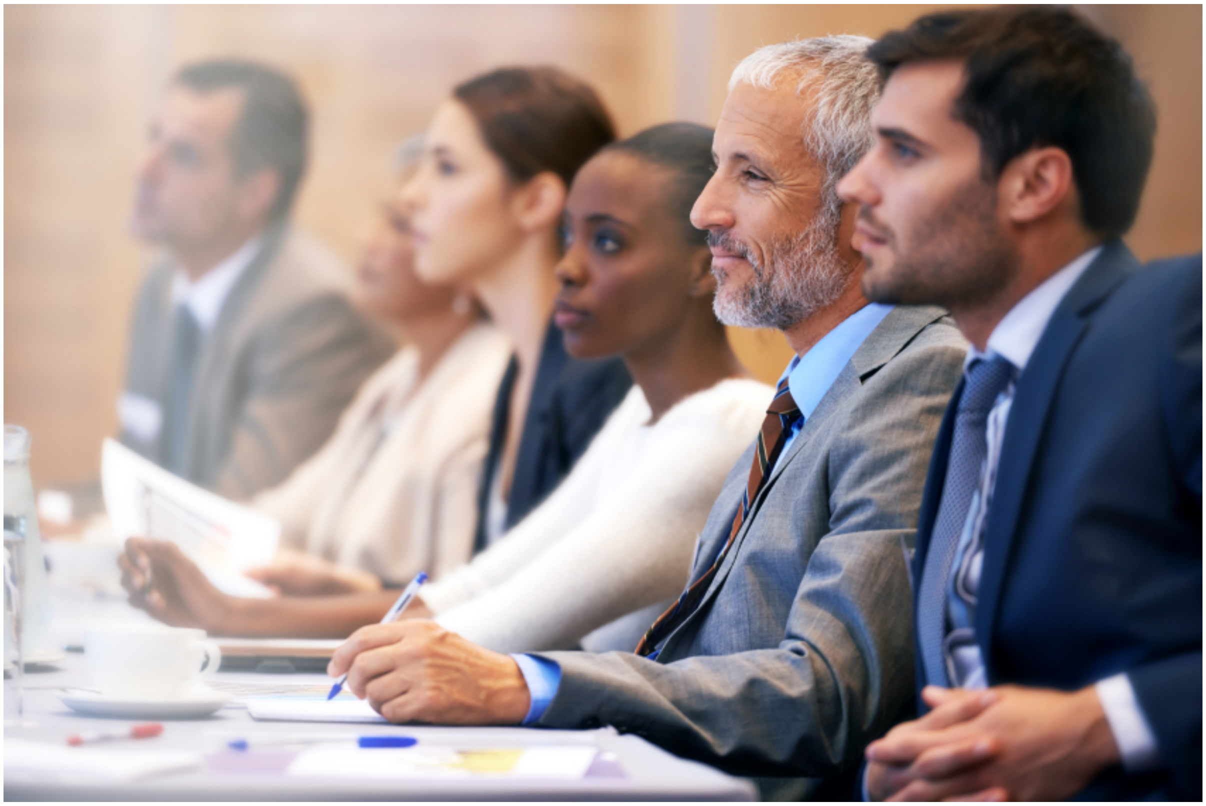

Questions to consider

Overarching question to consider

- What would make decision-making processes for cancer-drug funding trustworthy?

Additional questions to consider

- Who should be included in decision-making processes?
- What should the role of citizens be in decision-making processes?
- What should the role of people living with or affected by cancer be in decision-making processes?

Element 3 – Acting on decisions to achieve impact

Overview
The focus of this element is on identifying what citizens think would make actions taken on decisions to be transparent and trustworthy. Evidence and questions to consider during your deliberations are provided below.

Evidence to consider
Systematic reviews of different approaches to providing access to prescription drugs have found that:

- greater drug coverage reduces use of other healthcare services;(30)
- shifting costs to the patient may reduce government drug costs but may also lead to worse health outcomes and more use of other services;(30;31)
- prior approval (e.g., where a physician applies on behalf of the patient) for the use of some drugs can reduce costs and improve their use;(32) and
- setting a single price for all drugs in a category (e.g., drugs to treat a specific condition) has decreased drug prices and expenditures by insurers and patients, as well as increased the use of appropriate medications (but not increased the use of other medical services).(33)

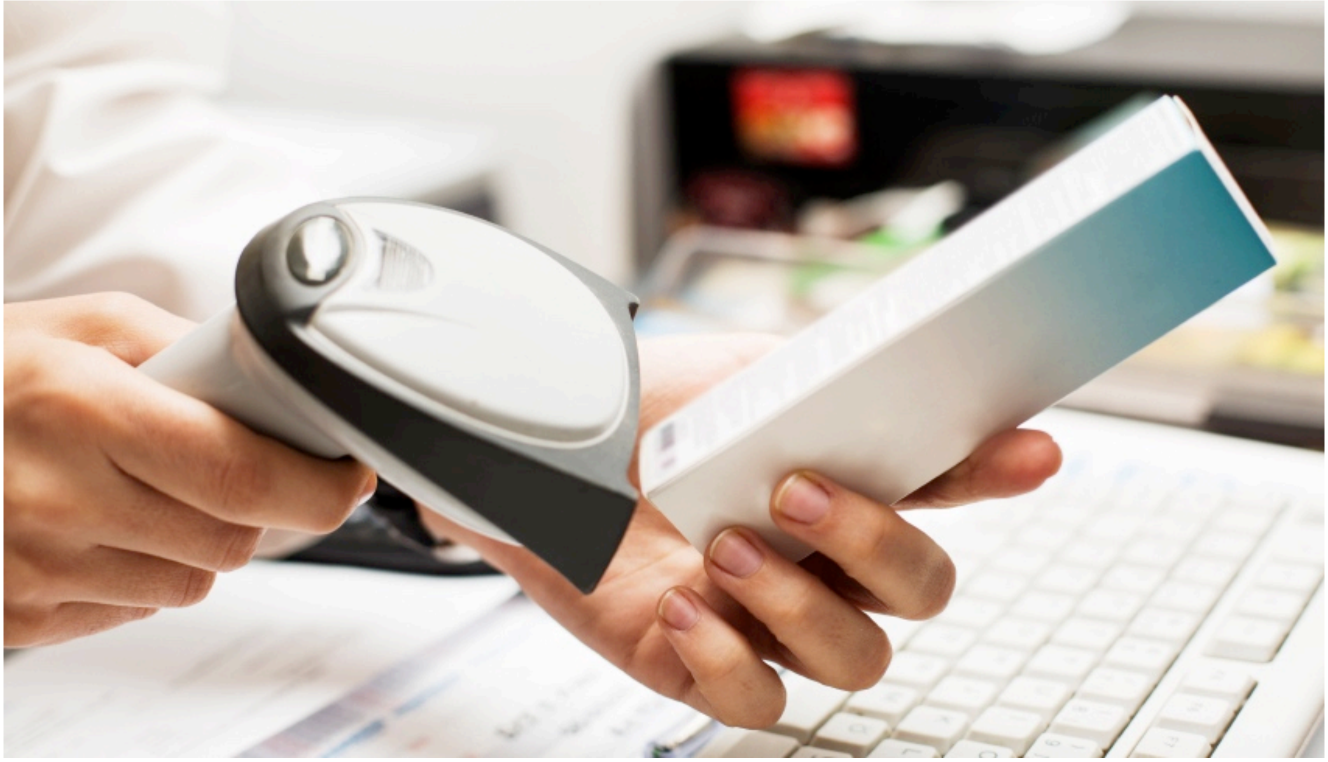

Questions to consider

Overarching question to consider

- What would make approaches to acting on decisions about funding cancer drugs trustworthy?

Additional questions to consider

- Under what conditions should expensive drugs be paid for when doctors and patients want them, even if there is little evidence of health benefit?
- Should we have cancer-specific drug formularies or one formulary for all drugs?
- Should governments require private plans to have a cancer drug formulary?
- What would make compassionate access programs for drugs more trustworthy?
- Should there be a limit to compassionate access programs?

Implementation considerations

It is important to consider potential barriers for implementing the proposed elements of an approach to address the problem. These barriers may affect different groups (e.g., patients, citizens, healthcare providers), different healthcare organizations or the health system. While some barriers could be overcome, others could be so substantial that they force a re-evaluation of whether an element should be pursued in a particular way. Some potential barriers to implementing the elements could include:

- provincial and territorial health-system leaders may have different readiness for change and collaboration (although as noted below under windows of opportunity, there is increasing interest in pan-Canadian leadership);
- groups (e.g., patient groups, disease-specific groups or pharmaceutical industry) mobilizing to support approval and funding for certain drugs and/or to oppose decisions to not fund certain drugs;
- public concern about why funding decisions for cancer drugs are treated differently than other drugs; and
- different groups (e.g., policymakers, clinicians and/or pharmaceutical companies) resisting the removal of compassionate access programs that give a way to fund drugs on a one-time basis to avoid unpopular decisions (e.g., because they don't want to be seen as saying no to cancer patients), or to retain market access for some drugs.

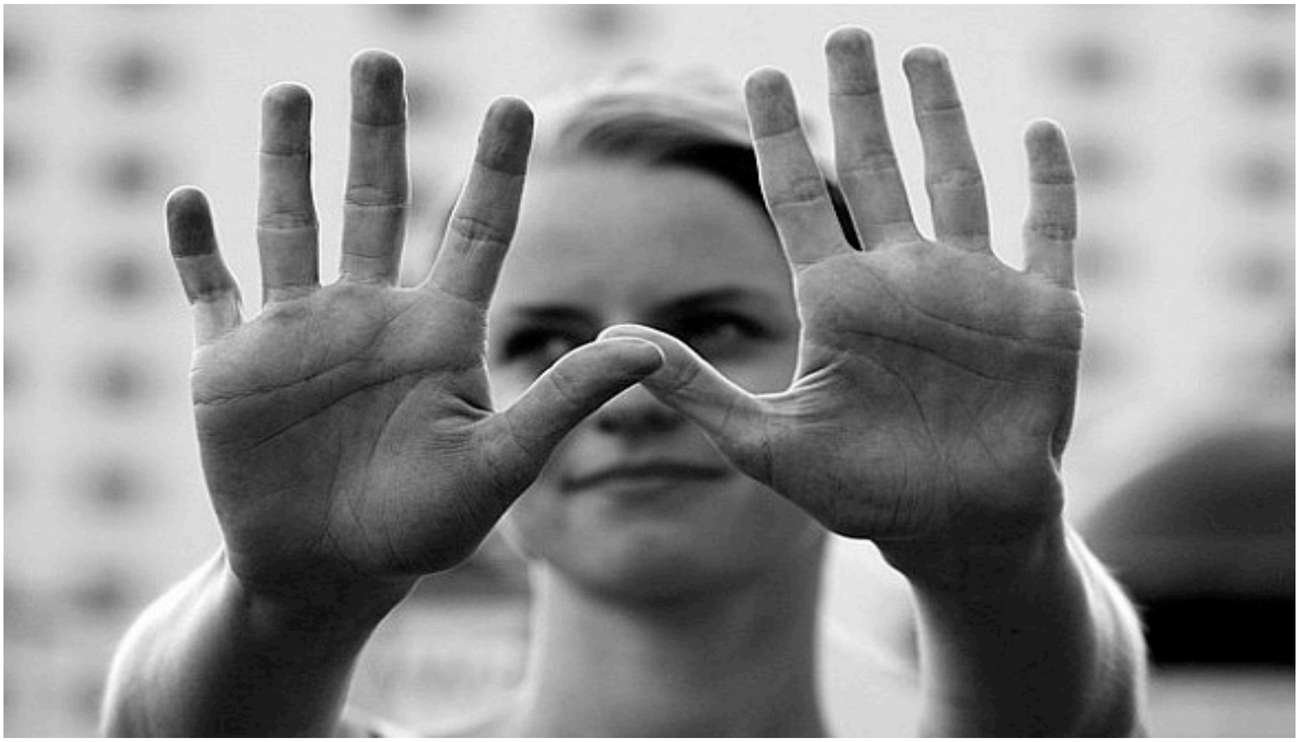

The implementation of the elements could also be influenced by the ability to take advantage of potential windows of opportunity. This could be, for example, a recent event that was highly publicized in the media, a crisis, a change in public opinion, or an upcoming election. A window of opportunity can facilitate the implementation of an element.

Examples of potential windows of opportunity

- **Interest in pan-Canadian leadership:** Following the election of a new federal government, collaboration among federal, provincial and territorial governments appears to have new momentum. Also, the use of evidence is now being prioritized in all of the thinking at the federal level, and a pan-Canadian bargaining process is already generating greater value for money in price negotiation.
- **Difficult economic times:** Sometimes difficult economic situations force the development of innovative policy approaches for making tough decisions.
- **Public interest:** The public is increasingly wanting to have a voice in decisions that affect them, coupled with there being increasingly better ways to do this.

In considering these potential barriers and windows of opportunity, recall the questions we posed at the beginning of the brief, which we have provided in Box 6 below.

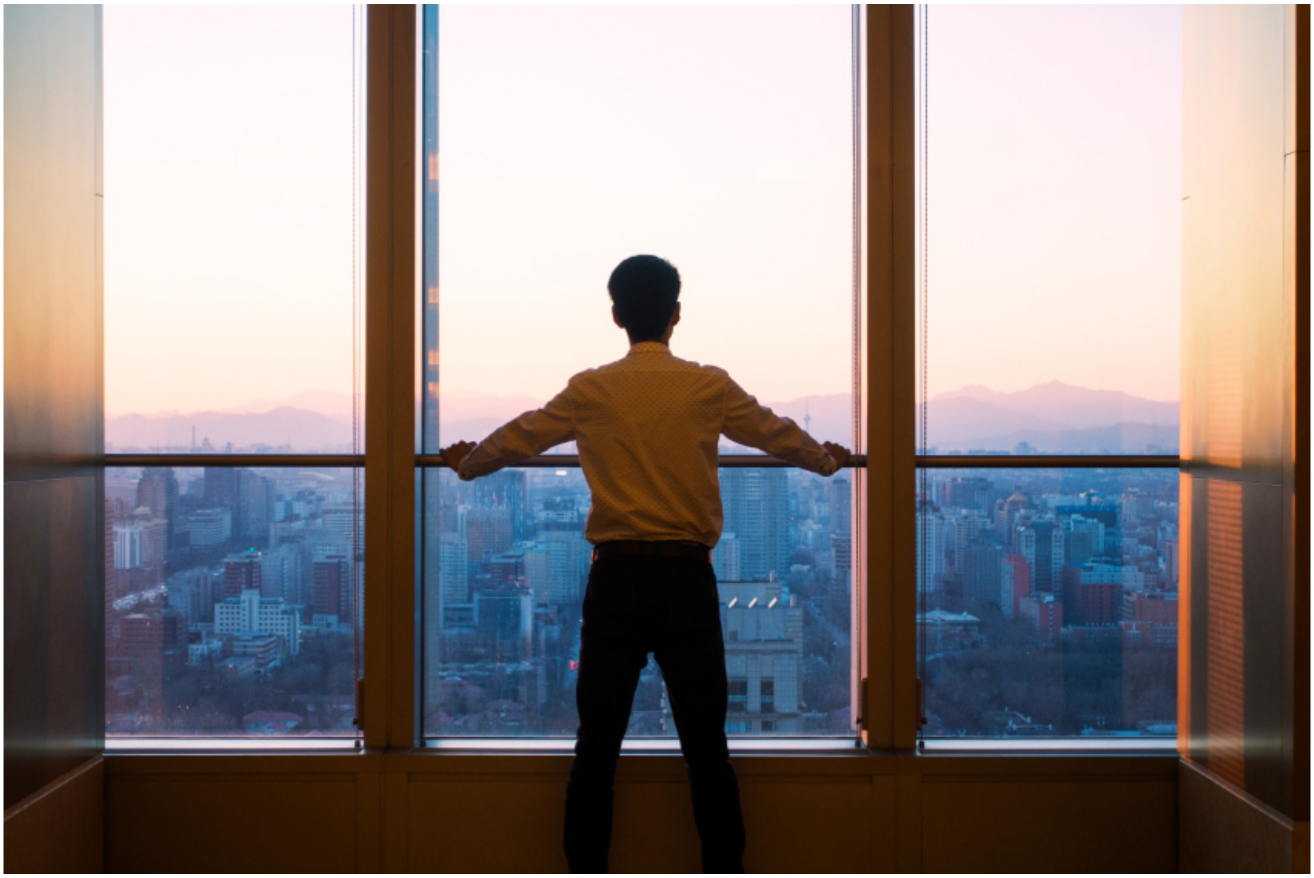

Box 6: A reminder of the questions to consider for your deliberations

Overarching question to consider

- How can funding decisions about cancer drugs be made in a way that is fair and sustainable?

Additional questions (please also see those provided as part of each element)

- How should we approach making decisions to fund new cancer drugs?
- How should we approach making decisions to stop or limit funding for some cancer drugs now being used?
- What would make you trust the decisions that are made about the funding of cancer drugs, or for actions taken to implement these decisions?
- Should drug funding decisions for cancer be treated differently than funding decisions for other drugs? Why?

Acknowledgments

Authors

Michael G. Wilson, PhD, Assistant Director, McMaster Health Forum, and Assistant Professor, McMaster University
John N. Lavis, MD, PhD, Director, McMaster Health Forum, and Professor, McMaster University
Julia Abelson, PhD, Professor, McMaster University
Michael Burgess, PhD, Professor, University of British Columbia
Stuart Peacock, DPhil, Co-Director, Canadian Centre for Applied Research in Cancer Control (ARCC) and Professor, Simon Fraser University
Laura Tripp, M.Sc., Research Coordinator, McMaster University
Colene Bentley, PhD, Health Services Researcher, Canadian Centre for Applied Research in Cancer Control (ARCC)

Funding

The citizen brief and the citizen panel it was prepared to inform were funded by the Canadian Partnership Against Cancer. The McMaster Health Forum receives both financial and in-kind support from McMaster University. ARCC is funded by the Canadian Cancer Society Research Institute (#2015-703549). The views expressed in the evidence brief are the views of the authors and should not be taken to represent the views of the funders.

Conflict of interest

The authors declare that they have no professional or commercial interests relevant to the citizen brief. The funder played no role in the identification, selection, assessment, synthesis, or presentation of the research evidence profiled in the citizen brief.

Merit review

The citizen brief was reviewed by a small number of citizens, other stakeholders, policymakers and researchers in order to ensure its relevance and rigour.

Acknowledgments

The authors wish to thank the entire McMaster Health Forum and the ARCC teams for support with project coordination, as well as for the production of this citizen brief. We are grateful to Steering Committee members (Mary Argent-Katwala, Brent Fraser and Suzanne McGurn) and merit reviewers (Marjorie Morrison and Terry Sullivan) for providing feedback on previous drafts of this brief. The views expressed in this brief should not be taken to represent the views of these individuals.

Citation

Wilson MG, Lavis JN, Abelson J, Burgess M, Peacock S, Tripp L, Bentley C. Citizen Brief: Making Fair and Sustainable Decisions about Funding for Cancer Drugs in Canada. Hamilton, Canada: McMaster Health Forum, 30 October

ISSN

2292-2334 (Online)

References

1. Ontario Ministry of Health and Long-Term Care. Ontario Public Drug Programs. Ontario Ministry of Health and Long-Term Care 2016 June 7.
2. Buist S. New cancer drugs raise hope for patients, but carry steep costs. Toronto Star and Hamilton Spectator 2016 February 28;Available from: URL: http://www.thestar.com/news/insight/2016/02/28/medical-advances-to-extend-cancer-survival-rates-outpacing-our-ability-to-pay-for-treatments.html
3. Province of Nova Scotia. Nova Scotia Pharmacare. Province of Nova Scotia 2016 April 25;Available from: URL: http://novascotia.ca/dhw/pharmacare/
4. CanEngage. Making Decisions About Funding for Cancer Drugs: A Deliberative Public Engagement. Vancouver, Canada: CanEngage.ca; 2014.
5. Mayo Clinic. Chemotherapy. Mayo Clinic 2016 March 15;Available from: URL: http://www.mayoclinic.org/tests-procedures/chemotherapy/basics/definition/prc-20023578
6. Saskatchewan Cancer Agency. Who Pays for Cancer Drugs in Saskatchewan? Saskatchewan Cancer Agency 2016 April 25;Available from: URL: http://www.saskcancer.ca/Default.aspx?DN=04763ac0-9c06-49d1-a79e-3389ae36a6fe
7. Régie de l'Assurance Maladie Québec. Amount to pay for prescription drugs. Government du Québec 2016 May 9;Available from: URL: http://www.ramq.gouv.qc.ca/en/citizens/prescription-drug-insurance/Pages/amount-to-pay-prescription-drugs.aspx
8. Régie de l'Assurance Maladie Québec. Prescription Drug Insurance - Eligibility. Government du Québec 2016 May 9;Available from: URL: http://www.ramq.gouv.qc.ca/en/citizens/prescription-drug-insurance/Pages/eligibility.aspx
9. Mayo Clinic. Biological therapy for cancer. Mayo Clinic 2016 March 15;Available from: URL: http://www.mayoclinic.org/tests-procedures/biological-therapy-for-cancer/basics/definition/prc-20022365
10. Government of Saskatchewan. Extended Benefits and Drug Plan. Government of Saskatchewan 2016 April 25;
11. Health Canada. How Drugs are Reviewed in Canada. Health Canada 2016 March 14;Available from: URL: http://www.hc-sc.gc.ca/dhp-mps/prodpharma/activit/fs-fi/reviewfs_examenfd-eng.php

12. No authors listed. The World Health Organization Quality of Life Assessment (WHOQOL): development and general psychometric properties. Social Science and Medicine 1998;46(12):1569-85.

13. Canadian Partnership Against Cancer, The pan-Canadian Oncology Drug Review. How Cancer Drug Funding Decisions are Made. Canadian Partnership Against Cancer and the pan-Canadian Oncology Drug Review 2013;Available from: URL: https://www.ccra-acrc.ca/images/CommunityForum/PrePARE-how-cancer-drug-funding-decisions-are-made.pdf

14. Canadian Cancer Society's Advisory Committee on Cancer Statistics. Canadian Cancer Statistics 2015. Toronto, Canada: Canadian Cancer Society; 2015.

15. Statistics Canada. Leading Causes of Death, by Sex. Statistics Canada 2015 December 10;Available from: URL: http://www.statcan.gc.ca/tables-tableaux/sum-som/l01/cst01/hlth36a-eng.htm

16. Centre for Chronic Disease Prevention PHAoC. Chronic Disease and Injury Indicator Framework: Quick Stats 2015 edition. Ottawa, Canada: Public Health Agency of Canada; 2015.

17. Public Health Agency of Canada. Economic Burden of Illness in Canada. Public Health Agency of Canada 2014 June 20;Available from: URL: http://www.phac-aspc.gc.ca/ebic-femc/index-eng.php

18. de Oliveira C, Bremner KE, Pataky R, Gunraj N, Haq M, Chan K et al. Trends in use and cost of initial cancer treatment in Ontario: A population-based descriptive study. CMAJ Open 2013;1(4):E151-E158.

19. Canadian Institute for Health Information. National Healt Health Expenditure Trends. Ottawa, Canada: Canadian Institute for Health Information; 2015.

20. OECD. Health at a Glance 2015: OECD Indicators. Paris, France: OECD Publishing; 2015.

21. Canadian Institute for Health Information. Drivers of Prescription Drug Spending in Canada. Ottawa, Canada: Canadian Institute for Health Information; 2012.

22. Canadian Institute for Health Information. Drivers of Prescription Drug Spending in Canada, 2013. Ottawa, Canada: Canadian Institute for Health Information; 2015.

23. Cancer Care Ontario, Ontario Ministry of Health and Long-Term Care. Growth in Ontario Cancer Spending FY95/95 to FY15/16. Toronto, Canada: Cancer Care Ontario & Ontario Ministry of Health and Long-Term Care; 2016.

24. CanCertainty. Cost of Same Cancer Treatment by Province. CanCertainty 2016;Available from: URL: http://www.cancertaintyforall.ca/cost_infographic

25. Longo CJ, Deber R, Fitch M, Williams AP, D'Souza D. An examination of cancer patients' monthly 'out-of-pocket' costs in Ontario, Canada. European Journal of Cancer Care 2016;16(6):500-7.
26. Nelson C. The Financial Hardship of Cancer in Canada. Winnipeg, Canada: Canadian Cancer Society, Manitoba Division; 2010.
27. Taylor DW. Benefits outweigh costs in universal healthcare: Business case for reimbursement of take-home cancer medicines in Ontairo and Atlantic Canada. American Journal of Medicine and Medical Sciences 2014;4(4):126-38.
28. Andrews J, Guyatt G, Oxman AD, Alderson P, Dahm P, Falck-Ytter Y et al. GRADE guidelines: 14. Going from evidence to recommendations: the significance and presentation of recommendations. Journal of Clinical Epidemiology 2013;66(7):719-25.
29. Fretheim A, Schunemann HJ, Oxman AD. Improving the use of research evidence in guideline development: 3. Group composition and consultation process. Health Res Policy Syst 2006;4:15.
30. Kesselheim AS, Huybrechts KF, Choudhry NK, Fulchino LA, Isaman DL, Kowal MK et al. Prescription drug insurance coverage and patient health outcomes: A systematic review. American Journal of Public Health 2015;105(2):e17-e30.
31. Luiza VL, Chaves LA, Silva RM, Emmerick-Isabel CM, Chaves GC, Fonseca de Araújo SC et al. Effects of cap and co-payment on rational use of medicines. Cochrane Database of Systematic Reviews 2015;(5):CD007017.
32. Green CJ, Maclure M, Fortin PM, Ramsay CR, Aaserud M, Bardal S. Pharmaceutical policies: Effects of restrictions on reimbursement. Cochrane Database of Systematic Reviews 2010;(8):CD008654.
33. Lee JL, Fischer MA, Shrank WH, Polinski JM, Choudhry NK. A systematic review of reference pricing: Implications for US prescription drug spending. Am J Manag Care 2012;18(11):e429-e437.

McMaster University

McMaster HEALTH FORUM

>> Contact us

McMaster Health Forum
1280 Main St. West, MML-417
Hamilton, ON Canada L8S 4L6
Tel: +1.905.525.9140 x 22121
Email: mhf@mcmaster.ca

>> Follow us

mcmasterhealthforum.org
healthsystemsevidence.org
healthsystemslearning.org

tinyurl.com/mhf-YouTube
tinyurl.com/mhf-Facebook
tinyurl.com/mhf-Twitter

EVIDENCE >> INSIGHT >> ACTION
