# Supplementary material for: Addressing the affordability of cancer drugs: using deliberative public engagement to inform health policy
Source: Health Res Policy Syst. 2019 Feb 7;17:17. doi: 10.1186/s12961-019-0411-8 (PMC6367823; doi:10.1186/s12961-019-0411-8)
Supplement: Supplementary file 2 — List of recommendations by province and deliberative topic. (PDF 824 kb) [file 12961_2019_411_MOESM2_ESM.pdf]

Additional file 2: List of Recommendations by Province and Deliberative Topic

Notes

1. The recommendations appear in the order in which they were generated by participants at each panel, with some re-organizing to align them more closely with the relevant deliberation topics. They are numbered for ease of reading only and do not suggest equivalencies across panels.

2. The following terms are used to indicate the degree of support for each recommendation: "All support" indicates that all participants agreed with the recommendation; "Support" indicates that a majority of participants agreed with the recommendation; "Persistent disagreement" indicates that participants were deeply divided about the recommendation.

3. For Ontario Recommendation 4 and Nova Scotia Recommendation 4, participants voted for one of several options. Here "support" indicates that some participants supported the option.

TOPIC 1: WHAT SHOULD GUIDE POLICY DECISIONS ABOUT WHETHER TO FUND NEW CANCER DRUGS, OR CHANGE THE FUNDING PROVIDED FOR EXISTING CANCER DRUGS?

Saskatchewan	Ontario	Quebec – English	Quebec – French	Nova Scotia
**Recommendation 1**<br>There should be a decision-making tool that evaluates and ranks drugs against identifiable criteria (Support)	**Recommendation 1**<br>Consider the quality of life during the remaining years, regardless of the patient's age (Support)	**Recommendation 1**<br>Consider the quality of life for the remaining years of life regardless of the patient's age (Support)	**Recommendation 1**<br>Funding for cancer drugs should not be discriminatory (All support)	**Recommendation 1**<br>When we are making recommendations, we take into account some factors: we all agree that prevention is of utmost importance (All support)
**Recommendation 2**<br>The criteria used in the decision-making tool should include but are not limited to: cost, quality and quantity of life, side effects, effectiveness, accessibility, incidence and type of cancer, mortality, age, duration of treatment, sustainability of the drug (uninterrupted supply), and ability to compare to other provinces' decisions (Support)	**Recommendation 2**<br>Drugs that increase the chance of full recovery should be prioritized (curative) (Persistent disagreement)	**Recommendation 2**<br>Policy makers should try to find a balance between quality of life and quantity of life (Support)	**Recommendation 2**<br>A significant improvement in the quality of life must justify a significant increase in the cost of drugs (Support)	**Recommendation 2**<br>There should be baseline criteria for funding any drug (All support)
**Recommendation 3**<br>Doubling the cost of a drug is worth it if it means patients can go from being dependent to being independent with improved mental health outcomes (Support)	**Recommendation 3**<br>Drugs that increase the chance of full recovery should be prioritized (i.e., drugs that are used to try to cure cancer patients should be given higher priority than drugs that are used for terminally ill cancer patients (Persistent disagreement)	**Recommendation 3**<br>Doubling the cost of a drug is worth it if it means that patients can go from being dependent to being independent. Being independent lowers the costs elsewhere in the system and beyond (All support)	**Recommendation 3**<br>If a new cancer drug is more costly, it should minimally increase the duration of life and improve the quality of life (Support)	**Recommendation 3**<br>Baseline criteria for funding any cancer drug should include moderate improvements to at least one of: quality of life, length of life, cost effectiveness (Support)

**Recommendation 4**<br>When doubling the cost of a drug, there needs to be at least 12 months of additional duration of life (Support)	**Recommendation 4**<br>When doubling the cost of a drug, there needs to be X months of additional duration of life*<br>*Note: in reference to a scenario that allowed participants to consider different options for duration of life<br>a. 3 months (Support)<br>b. 6 months (Support)<br>c. 12 months (Support)<br>d. 18 months (Support)<br>e. 24 months (Support)	**Recommendation 4**<br>We should prioritize cancer drugs that are easier to access in remote locations, as long as the medications are of comparable effectiveness (Support)	**Recommendation 4**<br>The government should not disinvest in cancer drugs (Support)	**Recommendation 4**<br>The most important criterion to consider when funding cancer drugs is:<br>a. Quality of life (Support)<br>b. Length of life (Support)<br>c. Cost-effectiveness (No support)<br>d. Abstain (Support)
**Recommendation 5**<br>Even if a drug offers slightly less quality of life and quantity of life, if it saves any amount of money it's justified to accept it (Support)	**Recommendation 5**<br>Decision makers need access to the best available comprehensive data on how effective the drug is and what are the effects on other parts of the health system when making policy choices (opportunity costs) (All support)	**Recommendation 5**<br>When there isn't a lot of difference between two drugs, we prefer any amount of savings provided the savings are reinvested in the drug funding budget (Support)	**Recommendation 5**<br>If the saving realized by a change in a drug allows for investment in cancer research, this change is justified (Support)	**Recommendation 5**<br>We should discontinue funding a cancer drug when there is another drug available of comparable effectiveness and less cost (Support)
**Recommendation 6**<br>When considering a new drug, the age of the patient group should not be a factor (Support)	**Recommendation 6**<br>Even if a drug offers slightly less quality of life and quantity of life but is still comparable, if it saves any amount of money it's justified to select it (Support)	**Recommendation 6**<br>Drugs should be re-reviewed based on evidence of alternative drugs already in use and also considering patient experiences (All support)	**Recommendation 6**<br>We should implement a usable database – mandatory and confidential, and updated by physicians – accessible to researchers and, within certain limits, decision makers allowing them to have a clear picture of patients' cancer journey (previous characteristics, cancer types, medications or interventions, short- to long-term clinical effects) (Support)	**Recommendation 6**<br>Evidence of effectiveness must be based on full disclosure to the regulator of clinical trial sample characteristics, full data sets, and should be peer reviewed (Support)
	**Recommendation 7**<br>When we consider new drugs, we need to consider the costs and benefits of existing drugs and, if needed, to delist the existing ones (grandfathering allowed) (Support)			**Recommendation 7**<br>When we consider new drugs, we need to consider the costs and benefits of existing drugs and, if needed, to delist the existing one (grandfathering allowed) (All support)

	**Recommendation 8**<br>When we fund new drugs we should be clearer about what the public is willing to pay for minimal improvements in survival, even though this may mean saying no to some new cancer drugs (Support)			**Recommendation 8**<br>Life extension is valuable provided there is a reasonable quality of life (Support)
	**Recommendation 9**<br>Drugs should not be funded unless we have strong evidence (e.g., a well designed clinical trial) that they are effective, even though this means some patients may not get the new drug until the evidence becomes stronger (Persistent disagreement)			

TOPIC 2: WHAT WOULD MAKE CANCER DRUG FUNDING DECISIONS TRUSTWORTHY?

Saskatchewan	Ontario	Quebec – English	Quebec – French	Nova Scotia
**Recommendation 7**<br>The criteria used within the [decision-making] tool are important because [they] facilitate decision making that is sustainable, defensible, transparent, objective, accountable, and fair (Support)	**Recommendation 10**<br>People need to know where to get reliable information about drug funding decisions (All support)	**Recommendation 7**<br>Pharmaceutical companies should not be involved in decisions to fund cancer drugs (All support)	**Recommendation 7**<br>Decision-making processes must be made transparent; a wide range of mechanisms should be used for informing and communicating (in an accessible language), the various options available during the decision-making process, the choices made, and their effects (All support)	**Recommendation 9**<br>Processes used to make drug funding decisions should be transparent so the public understands how decisions are made and who is making them (Support)
**Recommendation 8**<br>Drug decision makers should receive broad, diverse public input as part of the information considered in their decisions (Support)	**Recommendation 11**<br>Processes used to make drug funding decisions should be transparent so the public understands how decisions are made (Support)	**Recommendation 8**<br>There should be a panel that includes input from stakeholders (including but not limited to policy makers, long-term care workers, patient advocates, financial analysts, oncologists, healthcare providers, researchers, pharmacists) directly related to the cancer drug that is being considered for funding* (Support)	**Recommendation 8**<br>Decision-making processes must include the participation of: public decision makers, health professionals, organizations against cancer, patient associations and citizens. Pharmaceuticals must not be part of this process (Support)	**Recommendation 10**<br>Trustworthy drug funding decisions should NOT require patient members (Persistent disagreement)

		*Note: See recommendation 11 about pharmaceutical companies		
**Recommendation 9**<br>Drug decisions and decision processes must be transparent and publicly accessible (All support)	**Recommendation 12**<br>Is it appropriate to consider jobs and the economy when making drug funding decisions? (Persistent disagreement)	**Recommendation 9**<br>There should be more public awareness and transparency around the cancer drug approval process, especially with respect to Bill 28 and where to address concerns or questions (Support)	**Recommendation 9**<br>Ensure that members on the decision-making committee are not in conflict of interest. Limiting the duration of mandate could contribute to this objective (Support)	**Recommendation 11**<br>Trustworthy drug funding decisions should NOT require members of the general public as participants (Persistent disagreement)
**Recommendation 10**<br>People involved in making drug funding decisions should be screened so they act in the interest of the public, not of themselves (All support)	**Recommendation 13**<br>All bodies that oversee drug funding decisions should have members who represent lots of different perspectives, e.g., industry, patients, public, oncologists, academics, and policy makers (Support)		**Recommendation 10**<br>It is important to have a process for regularly replacing members of decision-making committees to ensure the contribution of new perspectives and ideas (2 consecutive mandates of 4 years with overlap) (Support)	**Recommendation 12**<br>The public and public values should play a role in cancer drug funding decisions and this should happen in different ways and at different times – e.g., public sitting on funding committees, citizens panels, etc. (Support)
**Recommendation 11**<br>Educational materials and videos, like the ones used in this deliberation, should be available to the public, as appropriate (All support)	**Recommendation 14**<br>There should be an independent ombudsman for those who appeal decisions about funding for cancer drugs (Support)		**Recommendation 11**<br>The government must communicate in a transparent manner the rationale for its decisions to provide access to medications on an exceptional basis (Support)	

TOPIC 3: HOW CAN WE IMPROVE EXISTING APPROACHES TO DECISION MAKING ABOUT CANCER DRUG FUNDING?

Saskatchewan	Ontario	Quebec – English	Quebec – French	Nova Scotia
**Recommendation 12**<br>Provinces and regions should work towards a common drug formulary (Support)	**Recommendation 15**<br>Enhance access in remote communities by investing in portable drugs (i.e., oral instead of IV drugs) (Support)	**Recommendation 10**<br>There should be one cancer drug formulary that is common to all provinces (Support)	**Recommendation 12**<br>Nationalize the production of pharmaceutical products to save money as long as this does not impede access to all medications. We could target those where savings should be better (Support)	**Recommendation 13**<br>There should be a pan-Canadian approach to cancer drug funding decisions not just funding recommendations (All support)

**Recommendation 13**<br>If a drug is delisted, patients should be given the option to stay on that drug (grandfathering) while other alternatives are being considered (Support)	**Recommendation 16**<br>There should be a separate drug formulary for cancer drugs (Persistent disagreement)	**Recommendation 11**<br>The pharmaceutical companies should be required to release all clinical trial data on demand (Support)	**Recommendation 13**<br>Harmonize the purchase of drugs against cancer between provinces to achieve greater purchasing power (Support)	**Recommendation 14**<br>Approved drugs should be re-reviewed based on post-approval data (All support)
**Recommendation 14**<br>There should be a standard process to re-evaluate drugs already on the formulary to ensure their effectiveness. If they aren't effective, they should be delisted and the funds used elsewhere (Support)	**Recommendation 17**<br>Canadian provinces and territories should fund exactly the same bundle of cancer drugs (i.e., oral, IV and other cancer drugs), even though this may mean some provinces/territories will have to stop funding some drugs, while other provinces/territories will have to start funding some more drugs (Persistent disagreement)	**Recommendation 12**<br>Once a drug has been approved by the province, it should be communicated about and made accessible across the province as quickly as possible (All support)	**Recommendation 14**<br>Ensure the harmonization of drug formularies across Canada and between hospitals within the same province (Support)	**Recommendation 15**<br>How cancer drugs are administered should not restrict whether funding is provided for them (e.g., whether in the hospital or in the community) (Support)
			**Recommendation 15**<br>Approved drugs should be reviewed based on data – clinical and experiential – subsequent to their approval (and those data must be systematically collected) (All support)	**Recommendation 16**<br>Priority should be given to cancer drugs that improve access to treatments where access is poor (Support)
			**Recommendation 16**<br>Ensure that remote regions have access to the same treatments against cancer (All support)	
