# Supplementary material for: Addressing the affordability of cancer drugs: using deliberative public engagement to inform health policy
Source: Health Res Policy Syst. 2019 Feb 7;17:17. doi: 10.1186/s12961-019-0411-8 (PMC6367823; doi:10.1186/s12961-019-0411-8)
Supplement: Supplementary file 3 — List of recommendations from the pan-Canadian panel. (PDF 696 kb) [file 12961_2019_411_MOESM3_ESM.pdf]

Note: The following terms are used to indicate the degree of support for each recommendation: "All support" indicates that all participants agreed with the recommendation; "Support" indicates that the majority of participants agreed with the recommendation.

What are important features of a pan-Canadian approach to making funding decisions about cancer drugs?

Recommendation 1
There should be a mandatory pan-Canadian approach to cancer drug funding decisions (Support)

Recommendation 2
A re-evaluation process of the effectiveness of each drug that is funded is an important part of a pan-Canadian approach (Support)

Recommendation 3
Re-evaluated drugs that are found to be less effective than originally thought, or compared to alternatives, should be considered for delisting or reduced pricing (Support)

Recommendation 4
When there is scientific uncertainty there should be a regulated mechanism for compassionate access for funding for drugs outside of approved uses when recommended by the treating oncologist (Support)

Recommendation 5
Provinces and territories must all collaborate in pan-Canadian funding decisions (All support)

What are the trade-offs associated with a pan-Canadian approach to making funding decisions about cancer drugs?

Recommendation 6
Generally, if a pan-Canadian approach approves a drug for funding, if one can get it everybody gets it; if one doesn't, nobody does (Support)

Recommendation 7
Disinvesting* should be pursued as much as possible [while] giving patients and their oncologists the opportunity to stay on that drug (*see citizen brief for definition) (Support)

How might these trade-offs be addressed to produce trustworthy decisions?

Recommendation 8
Any individual with a conflict of interest, be it personal or professional, shall not sit on the committee (Support)

Recommendation 9
The committee shall undergo regular equity audits to ensure the needs of vulnerable populations are met (Support)

Recommendation 10
Membership of the pan-Canadian decision making body should include a health economist, a clinician, and a representative from each province and territory at minimum (Support)

Recommendation 11
The decision-making process and justification for funding a drug or disinvesting or not funding should be made public (All support)
